# Redirecting meiotic DNA break hotspot determinant proteins alters localized spatial control of DNA break formation and repair

**DOI:** 10.1093/nar/gkab1253

**Published:** 2021-12-30

**Authors:** Randy W Hyppa, Joshua D Cho, Mridula Nambiar, Gerald R Smith

**Affiliations:** Division of Basic Sciences, Fred Hutchinson Cancer Research Center, Seattle, WA 98109, USA; Division of Basic Sciences, Fred Hutchinson Cancer Research Center, Seattle, WA 98109, USA; Division of Basic Sciences, Fred Hutchinson Cancer Research Center, Seattle, WA 98109, USA; Division of Basic Sciences, Fred Hutchinson Cancer Research Center, Seattle, WA 98109, USA

## Abstract

During meiosis, DNA double-strand breaks (DSBs) are formed at high frequency at special chromosomal sites, called DSB hotspots, to generate crossovers that aid proper chromosome segregation. Multiple chromosomal features affect hotspot formation. In the fission yeast *S. pombe* the linear element proteins Rec25, Rec27 and Mug20 are hotspot determinants – they bind hotspots with high specificity and are necessary for nearly all DSBs at hotspots. To assess whether they are also sufficient for hotspot determination, we localized each linear element protein to a novel chromosomal site (*ade6* with *lacO* substitutions) by fusion to the *Escherichia coli* LacI repressor. The Mug20-LacI plus *lacO* combination, but not the two separate *lac* elements, produced a strong *ade6* DSB hotspot, comparable to strong endogenous DSB hotspots. This hotspot had unexpectedly low *ade6* recombinant frequency and negligible DSB hotspot competition, although like endogenous hotspots it manifested DSB interference. We infer that linear element proteins must be properly placed by endogenous functions to impose hotspot competition and proper partner choice for DSB repair. Our results support and expand our previously proposed DSB hotspot-clustering model for local control of meiotic recombination.

## INTRODUCTION

The formation of viable haploid gametes from diploid precursor cells occurs during the two specialized nuclear divisions called meiosis. A critical event is the segregation of the parental centromeres, with their attached chromosomal arms, on the two replicated chromosomes (homologs) at the first division. Successful segregation requires in most species physical connection of the homologs by one or more crossovers in the arms (1,2). In conjunction with sister chromatid cohesion, a crossover provides tension during meiosis I to allow proper segregation; tension signals that the centromeres are going to opposite poles of the cell (3), as required for successful meiosis. Crossovers are formed by homologous genetic recombination between parental chromosomes. Crossing-over also generates genetic diversity among the progeny and thus aids evolution. These dual roles of crossing-over likely account for the nearly universal occurrence of recombination at high level during meiosis. Understanding meiotic recombination requires knowledge of its molecular determinants and their functions, the subject here.

DNA double-strand breaks (DSBs) occur at high frequency during meiosis, and their repair produces the crossovers critical for meiosis (4). DSBs are not uniformly distributed across the chromosomes. Rather, there are sites, called DSB hotspots, at which DSBs occur preferentially (5). In the fission yeast *Schizosaccharomyces pombe*, studied here, DSBs occur at hotspots up to 200 times more frequently than the genome median (6,7). Hotspots have also been characterized in the distantly related budding yeast *Saccharomyces cerevisiae*, which have hotspots up to 200 times the genome median (8) and in mice (up to 500 times the genome median (9) (reviewed in (5)). The determinants of DSB hotspots are not completely known in any case, but multiple factors, both DNA sequence and chromosome-bound proteins, contribute. The binding of certain transcription factors is a major determinant in some cases, but the individual transcription factors tested appear to account for only a small minority of hotspots across the genome (8,10). More general aspects of chromatin structure are also important.

Hotspots show a preference for nucleosome-depleted regions in *S. cerevisiae* (8,11,12) but less so in *S. pombe* (7,13). Histone modifications, such as histone H3 lysine 4 methylation (H3 K4Me), are correlated with DSB formation at most hotspots in *S. cerevisiae* and mice (14–16). In *S. pombe* acetylation of histone H3 lysine 9 (H3 K9Ac) is elevated at DSB hotspots (17), and the histone variant H2A.Z is needed to localize DSB-forming proteins to hotspots (18). Thus, a complex interplay of protein binding and chromatin structure appears to determine the distribution of DSBs across the genome.

DSB hotspots do not act independently—they interact with neighboring hotspots. Introduction of a novel hotspot reduces DSB frequency at nearby hotspots. This feature, called DSB competition, acts primarily along one homolog (‘in *cis*’) and over ∼200 kb regions in *S. pombe* (19) and ∼70 kb in *S. cerevisiae* (20–23). In addition, the frequency of two DSBs on the same chromatid, one at each hotspot, is less than that expected from independence (*i.e*., the product of the individual frequencies). This feature, called DSB interference, also acts over ∼200 kb in *S. pombe* (19) and ∼70 kb in *S. cerevisiae* (24). A model for hotspot competition and DSB interference embodying clustering of nearby hotspots has been proposed and supported by a variation of the chromosome conformation capture (3C) method, which demonstrated 3D clustering of DSB hotspots over ∼200 kb regions (19). These authors proposed that DSB hotspot competition and interference might result from the same mechanism. Results reported here indicate that they are separable features, in agreement with DSB interference being dependent on the Tel1 DNA damage response protein kinase in *S. cerevisiae* (24), whereas competition is independent of Tel1, although its strength varies between hotspots (25). DSB competition has been proposed to be independent of DSB formation *per se* (26) and to result from competitive loading of factors for DSB formation (20,23,27,28).

A clear case of DSB hotspot determinants acting across the whole genome is provided by the linear element (LinE) proteins of *S. pombe*. LinE proteins are meiosis–specific and bind along the chromosomes and form long lines (LinE structures) visible by light and electron microscopy (6,29–31). LinE structures resemble the axial element precursors to the meiosis–specific synaptonemal complex (SC) of other species (32), which forms a regular structure along and between homologous chromosome pairs from one end to the other of the many species investigated (33). Four *S. pombe* LinE proteins have been identified by genetics and microscopy—Rec10, Rec25, Rec27 and Mug20 (34–36). These proteins appear to act as a complex, since where tested deletion of any one renders foci of the other LinE proteins undetectable by light microscopy, except for Rec10, which remains diffusely visible in the nucleus (31,34,35,37). Rec10 has limited amino acid similarity to *S. cerevisiae* Red1 (36), and Rec27 has limited amino acid similarity to the *Caenorhabditis elegans* SC protein SYP-2 (6). Mug20 appears similar to DDL-1, an SYP-2-interacting protein, although DDL-1 has not to our knowledge been reported to be in the SC (38). Both SC and LinE structures are dissociated by the chaotropic agent 1,6-hexanediol (31,39) [but see (40)]. Thus, the *S. pombe* LinEs have several functional properties of the SC of other species.

Three LinE proteins—Rec25, Rec27 and Mug20—are determinants of DSB hotspots in *S. pombe*. Whole-genome analysis shows that these proteins bind to hotspots with high specificity and abundance: at hotspots there is up to 80 times the genome-median protein density (6). LinE protein abundance at hotspots is highly correlated with the DSB frequency at hotspots (Pearson's correlation coefficient *r* = 0.79–0.88). In the absence of any one of these three proteins, DSB frequency at most hotspots is strongly reduced or undetectable. DSBs apparently remain in ‘cold regions’ between hotspots, because there is significant residual meiotic recombination in mutants lacking any one (or tested pair) of these proteins (34,35). By contrast, in the absence of Rec10, DSBs and recombination are not detectable above background levels (6,41). Rec10 also localizes to DSB hotspots (up to ∼3 times the genome median), but with less specificity as there are other sites of localization (6,42). Rec10 interacts with the other LinE proteins, cohesin, and the DSB-forming complex (the Spo11 homolog Rec12 and its half-dozen essential partners) (42–44).

Although these three LinE proteins bind DSB hotspots with high specificity, no simple DNA sequence is discernible within the hotspot region that might account for LinE protein specificity for binding hotspots. Their preferential binding may depend on a complex set of factors which in turn bind a complex set of DNA sequences. This outcome has precluded testing whether LinE protein binding to a chromosomal site is sufficient to create a DSB hotspot.

Consequently, we have used a different experimental approach to show that binding of a LinE protein indeed is sufficient to create a DSB hotspot. In addition, our results reported here reveal unexpected complexities in the behavior of a novel set of LinE protein-dependent DSB hotspots, which sheds light on the molecular mechanisms controlling DSB hotspot activity and recombination dependent upon DSBs at hotspots and their repair (19,35).

## MATERIALS AND METHODS

### Strains and genetic methods

**Figure 1.**
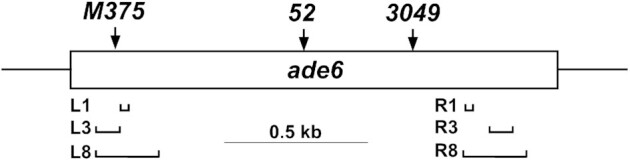
Location of *ade6* mutations. *lacO* operator arrays (horizontal brackets) of 1, 3 or 8 copies were substituted for an identical size interval of the *ade6* open reading frame (open box; 1659 bp) on the left (L) at the *ade6-M375* position (single bp control mutation G133T, three bp from the *ade6-M26* recombination hotspot with ATGACGT bound by the transcription factor Atf1-Pcr1), or on the right (R). The *ade6-52* single bp mutation (G796A), between the L and R arrays, was crossed with the arrays to determine intragenic recombinant frequencies. *ade6-3049* (C1214T) creates another ATGACGT heptamer and a strong DSB hotspot. *arg1*, 300 kb to the right, was used to determine intergenic recombinant frequencies. *bub1-243*, 0.65 kb to the left of *ade6*, and *vtc4-1104*, 1.49 kb to the right, were used for DNA analyses of recombinants and intermediates (Figure 6).

Genotypes and origins of *S. pombe* strains are in Supplementary Table S1. The notation ‘::’ indicates a substitution (deletion and insertion) of part or all of the gene to the left of the symbol with the gene to the right of the symbol; ‘:’ indicates a simple insertion (without deletion) near the gene to the left of the symbol. Growth media were described previously (45). Transformation of strains to introduce an allele into the chromosome used the lithium acetate method for homology-directed integration of polymerase chain reaction (PCR) products containing ∼80 bp of homology with the targeted gene (46); new chromosomal alleles were confirmed by sequencing a PCR product generated from the transformant. Oligonucleotides are in Supplementary Table S2, and plasmids are in Supplementary Tables S3 and S4. Genetic crosses to determine recombinant frequencies were done at 25°C on supplemented SPA medium (45). Intra- and inter-genic recombinant frequencies were determined by random spore analysis. Differential plating for total and recombinant (prototrophic) types was used to assay intragenic recombination between heterozygous *lacO* substitutions in *ade6*, such as *ade6-3101*, and *ade6-52* (primarily gene conversion); analysis of individual spore colonies by picking to grids and replica-plating was used to assay intergenic recombination between *ade6* and *arg1* (primarily crossing over) (see Figure 1).

### Generation of LinE-LacI fusion proteins

A Gibson Assembly Kit (New England Biolabs, Ipswich, MA) was used to create plasmids expressing the LinE-LacI fusion proteins. Two separate plasmids were used as templates for DNA synthesis; one contained the full-length LinE protein-coding DNA (including the 5′ and 3′ untranslated regions), and another (pRS406-CMV-LacI-NLS-3FLAG) contained the *lacI* gene followed by DNA encoding a nuclear localization signal (NLS; PKKKRKV) and three copies of the FLAG epitope (DYKDDDDK) for immunoprecipitation (47). To fuse LacI to a LinE’s C-terminus, the LinE-containing plasmid was linearized between its coding sequence and its translational stop codon. This procedure used inverse PCR with a forward primer that starts immediately after the stop codon and a reverse primer that starts immediately before the stop codon. In a parallel PCR, a linear fragment containing *lacI-nls-3FLAG* and its stop codon was generated with ∼20–25 extra nucleotides on both sides that were homologous to the region where *lacI* was to be inserted, adjacent to the LinE protein-coding sequence. Using Gibson Assembly, the two PCR products were assembled into a circular plasmid expressing LinE-LacI. To fuse LacI to a LinE’s N-terminus, the LinE protein-coding plasmid was linearized immediately before the start codon and combined, using Gibson Assembly, with a *lacI* fragment (without its stop codon) that had extra nucleotides homologous to the region of its insertion. Note that NLS-3FLAG is not present in LacI-Mug20 (*mug20-252*) or in Mug20-LacI' (*mug20-254*). DNA encoding a LinE fusion to LacI was placed on the chromosome by transformation to fluoro-orotic acid (FOA)-resistance of a *ura4* deletion strain containing the *ura4^+^* gene in place of the corresponding LinE protein-coding sequence at its endogenous locus (48,49).

### Generation of *lacO* arrays

*ade6* alleles with a single *lacO* were generated with the NEB Q5 mutagenesis kit and oligonucleotides containing *lacO* flanked by ∼20–25 *ade6* nucleotides separated by the size of the *lacO* insertion (27 bp), to form an exact substitution. *ade6* alleles with a short array of *lacO* were generated using Gibson Assembly from a plasmid containing *ade6* (such as pJC1 or pJC13) and a plasmid containing 3 or 8 copies of *lacO* repeats (pUC-TALO3 or pUC-TALO8, respectively) (47); each *lacO* repeat had the 27-bp *lacO* sequence 5′ ACTAGCAATTGTGAGCGGATAACAATT 3′. The *ade6* plasmid was linearized by inverse PCR, excluding the nucleotides in *ade6* to be substituted with *lacO* repeats. A parallel PCR generated a linear fragment containing the repeated *lacO* array and an additional 15 and 23 bp (L3 and L8) or 15 and 25 bp (R3 and R8) plus 15–27 *ade6* bp flanking the substitution site for homologous integration. The number of bp inserted equaled the number of *ade6* bp deleted, to maintain wt spacing of DNA flanking the *lacO* array. Using Gibson Assembly, the two PCR products were assembled into a circular plasmid containing *ade6* with *lacO* arrays. These alleles were placed on the chromosome by transformation to FOA-resistance of strain GP6104 (*ade6-3095::ura4^+^*, a substitution of the entire *ade6* ORF with *ura4^+^*). The nucleotide sequences of the *ade6* gene, wt and with each of the L1, L3, L8, R1, R3 and R8 substitutions, are in Supplementary Data following Supplementary Table S4. To couple the *ade6::lacO* substitutions with the flanking restriction site polymorphisms *bub1-243* (L) and *vtc4-1104* (R), strains GP8897 and GP8898 were similarly transformed. These strains, with flanking *bub1* or *vtc4* mutations, contain the *ade6-3103::ura4^+^* substitution (1.8 kb of *ura4^+^* DNA replacing the *ade6* ORF plus 201 bp 5′ and 78 bp 3′ of the ORF).

### Construction of *ura1::hphMX6*, *tel1::natMX6* and *mde2::hphMX6*

A DSB hotspot was introduced into the *ura1* gene by substituting bp –80–1607 of the *ura1* coding sequence with 1767 bp of the *hphMX6* hygromycin-resistance determinant in plasmid PCR2.1-hph (46,50). The PCR product using oligos OL4418 and OL4419 was used to transform strain GP9901 to hygromycin-resistance and uracil auxotrophy. The substitution *mde2::hphMX6* was made by generating a PCR product from plasmid PCR2.1-hph with OL4461 and OL4462 and transforming strain GP8875 to hygromycin-resistance. Strain GP8985 (containing the substitution *tel1::kanMX6*) was transformed to nourseothricin-resistance and kanamycin-sensitivity with a PCR product using oligos MD1 and MD2 and plasmid PCR2.1-nat (50).

### Meiotic induction and DNA preparation

Meiotic induction and DNA preparation were performed as described (51). Briefly, strains with *pat1-114* (temperature-sensitive) or *pat1-as1* (ATP analog-sensitive) were used to induce synchronous meiosis after nitrogen starvation (which produces G1-arrested cells) by shifting the temperature to 34°C for *pat1-114*, or by adding 3-MB-PP1 (Toronto Research Chemicals) to 25 μM at 25°C or 34°C for *pat1-as1*, and adding NH_4_Cl as a nitrogen source. All DNA analyses were done with cultures induced at 34°C except for those in Figure 4B at 25°C. Cells were harvested at the specified times after induction, embedded in agarose plugs, and digested with lytic enzymes to break the cells. The plugs were further treated with proteinase K, subsequently inactivated with phenylmethylsulfonyl fluoride (PMSF), and washed thoroughly with TE buffer.

Induction of meiosis was confirmed by flow cytometry to determine pre-meiotic DNA replication, which typically began at 2 h and was completed by 3 h at 34°C (3.5 and 5.5 h, respectively, at 25°C).

### DSB analysis

DNA in agarose plugs was digested with appropriate restriction enzymes and analyzed either by standard agarose gel electrophoresis (for fragments shorter than 20 kb) or pulsed-field gel electrophoresis (for fragments longer than 20 kb). Southern blot hybridization was performed as previously described (51). For the 150.5 kb *Sac*II fragment with *ade6*, DNA probes, made by PCR using oligos OL4416 and OL4417 and chromosomal DNA, correspond to positions 1427–1428 kb of chromosome III. Previously described were probes for the 11.8 kb *Bsr*GI fragment and the double-cut DNA fragment between *ade6* and a hotspot ∼75 kb to its right (49); for the 74.2 kb *Pme*I fragment containing *ade6* (6); and for the 501 kb *Not*I J fragment and the 64.4 kb *Pme*I fragment, both containing *mbs1* (52). See Supplementary Figure S1 for positions of restriction sites and probes. Signals were detected using a Typhoon Odyssey PhosphorImager system (GE Healthcare) and quantified using ImageQuant TL (GE Healthcare) software.

### DNA joint molecule and crossover analysis

DNA in agarose plugs was digested with *Bsr*GI and analyzed by two-dimensional (2D) gel electrophoresis to separate the branched DNA intermediates (joint molecules, such as Holliday junctions) at the *ade6* locus (51). Determination of inter-sister and inter-homolog Holliday junctions and crossover DNA fragments used heterozygous restriction sites for *Sca*I (*bub1-243*) and *Pml*I (*vtc4-1104*) flanking *ade6* and Southern blot hybridization using a probe for *ade6* previously described (53). Signals were detected as above.

### Statistics

Intragenic recombinant frequencies and DNA fragment data were expressed as mean ± standard error of the mean (SEM), based on n repeats and calculated using GraphPad software. Intergenic recombinant fractions were used to estimate one standard deviation (SD) based on the Poisson distribution (GraphPad); fractions were converted to centiMorgans (cM) using Haldane's relation (54). Two-sided unpaired *t* tests were used to evaluate statistical significance.

## RESULTS

### Chromosomal localization of Mug20-LacI fusion generates a strong recombination hotspot

Does forced localization of a LinE protein at a site in a non-hotspot region of the chromosome create a DSB hotspot; *i.e*., is LinE protein localization *sufficient* for hotspot determination? To answer this question, we used the *E. coli lacO*-LacI localization elements to target LinE-LacI fusion proteins to a *lacO* array introduced into the *S. pombe ade6* gene, which lacks noticeable DSB hotspots and has low-level recombination (55). We inserted *lacI-nls-3flag* at the C-terminus of the *rec10*, *mug20*, *rec25* and *rec27* coding sequences and determined the intragenic recombinant frequency between *ade6-52* and *ade6-3101*. The *ade6-3101* allele contains 8 copies of *lacO* substituted for an equal length of *ade6* near its left (5′) end, about 400 bp from *ade6-52*; this substitution covers the position of *ade6-M375* used as a non-hotspot control (Figure 1). In the absence of any fusion protein, the recombinant frequency was 89 Ade^+^/10^6^ viable spores (Table 1). There was an ∼2-fold or ∼1.5-fold increase in recombinant frequency when homozygous Rec10-LacI or Rec27-LacI, respectively, was present, and ∼40% decrease with homozygous Rec25-LacI. Strikingly, the Mug20-LacI fusion (*mug20-231*) gave an impressive 5- to 6-fold increase in recombinant frequency (to 530 Ade^+^/10^6^ viable spores) compared to wild type. Subsequent experiments used fusions of Mug20 and LacI.

**Table 1.** Mug20-LacI fusion is most active in creating a meiotic recombination hotspot

*ade6* allele	No fusion	Rec10-LacI (*rec10-233*)	Mug20-LacI (*mug20-231*)	Rec25-LacI (*rec25-230*)	Rec27-LacI (*rec27-232*)
*3101*	89 ± 13 (8)	209 + 28 (5)	529 ± 27 (8)	60 (1)*	158 ± 27 (5)

Intragenic recombinant frequency (Ade^+^ per 10^6^ viable spores) was assayed with *ade6-3101* and *ade6-52* in either the absence or presence of different LinE-LacI fusion proteins. *ade6-3101* contains 8 copies of the *lacO* operator substituted for part of the *ade6* gene (Figure 1). Data are mean ± SEM from (n) crosses. See Table S5 for data with additional *ade6::lacO* alleles.

*In independent experiments, the frequency was 70 ± 5 (*n* = 6) with Rec25-LacI and 119 ± 10 (*n* = 8) with Rec25.

In an attempt to get an even stronger recombination hotspot, we modified the organization of the *lacO* array by altering the number of *lacO* repeat units (1, 3 or 8) and by generating symmetrical LacI localization with direct repeat of the arrays on either the ‘left’ (5′ or ‘L’) or ‘right’ (3′ or ‘R’) side of *ade6*, or both (Figure 1). These double alleles are designated L3, R3; L3, R8; L8, R3 and L8, R8, where 3 and 8 indicate the number of direct *lacO* repeats (Supplementary Table S4). However, the largest increase among single-site substitutions was still obtained with the *ade6-3101* (L8) allele containing eight *lacO* copies on the left (Table 2). Double-site substitutions (*e.g*., L3 R3) produced a low frequency of recombinants (double exchange events), making them less useful to work with.

**Table 2.** Meiotic recombination hotspot requires both *lacO* and Mug20-LacI fusion

*ade6* allele (*lacO* operators)	No fusion (*mug20^+^*)	Mug20-LacI (*mug20-231*)	Fold increase by LacI fusion
*3098* (L1)	185 ± 35 (5)	369 ± 8 (3)	2.0
*3102* (L3)	121 ± 18 (5)	502 ± 82 (5)	4.1
*3101* (L8)	89 ± 13 (8)	529 ± 27 (8)	5.9
*3099* (R1)	87 ± 11 (5)	220 ± 4 (2)	2.5
*3111* (R8)	52 (1)	257 (1)	4.9
*3106* (L3, R3)	11 ± 1 (5)	97 ± 4 (5)	8.8
*3107* (L3, R8)	9 ± 1 (6)	52 ± 6 (6)	5.8
*3108* (L8, R3)	7 ± 1 (5)	68 ± 5 (5)	9.7
*3109* (L8, R8)	6 ± 1 (4)	31 ± 4 (4)	5.2
*M375* (none)	205 ± 9 (4)	208 ± 7 (4)	1.0

Intragenic recombinant frequency (Ade^+^ per 10^6^ viable spores) was assayed with *ade6* alleles bearing the indicated *lacO* array (Figure 1) and *ade6-52* in either the absence (*mug20^+^*) or presence of the Mug20-LacI fusion (*mug20-231*). ‘L’ and ‘R’ indicate left and right sides of *ade6*, where the *lacO* operators (number per array indicated) were positioned; *ade6-M375* is a single bp mutation near the position of the L arrays. Note that with double *lacO* substitutions, such as L3 R3, Ade*^+^* recombinants require double exchanges. Data are mean ± SEM from (*n*) crosses, or value or range for *n* = 1 or 2.

We also varied the position of the LacI fusion on the Mug20 protein. We tested LacI fusions on the N-terminus (LacI-Mug20; *mug20-252*) or the C-terminus (Mug20-LacI'; *mug20-254*) without the NLS (nuclear localization signal) and FLAG (immunoprecipitable) components in the *mug20-231* allele used above. There were modest increases in the recombinant frequency with the N-terminal fusion (LacI-Mug20; 8-fold higher than *mug20^+^*) or a C-terminal fusion without the NLS or 3FLAG (Mug20-LacI'; 6.5-fold), compared to Mug20-LacI (*mug20-231*; 6-fold) (Table 3). We used *mug20-252* (designated as LacI-Mug20 fusion) or *mug20-231* (Mug20-LacI fusion) and *ade6-3101* (L8 *lacO* array) for the remaining analyses.

**Table 3.** LacI fusion to the N-terminus of Mug20 creates the most active recombination hotspot

*ade6* allele	No fusion (*mug20^+^*)	Mug20-LacI (*mug20-231*)	LacI-Mug20 (*mug20-252*)	Mug20-LacI’ (*mug20-254*)
*3101*	89 ± 13 (8)	529 ± 27 (8)	713 ± 107 (5)	576 ± 106 (4)

Intragenic recombinant frequency (Ade^+^ per 10^6^ viable spores) was assayed with *ade6-3101* and *ade6-52* in either the absence or presence of the indicated LacI fusions with Mug20. *ade6-3101* contains 8 copies of the *lacO* operator substituted for part of the *ade6* gene (Figure 1).

Data are mean ± SEM from (*n*) crosses. See Table S6 for data with additional *ade6::lacO* alleles.

We further tested the specificity of recombination observed with *ade6-3101* and either the LacI-Mug20 or Mug20-LacI fusion by addition of isopropylthiogalactoside (IPTG) to disrupt the *lacO-*LacI interaction. Addition of IPTG during meiosis reduced the *ade6* recombinant frequency significantly only with the combination of *ade6-3101* and LacI-Mug20 fusion, from 820 to 94 Ade^+^ per million viable spores (Supplementary Tables 4A and S6). Intergenic recombination between *ade6* and *arg1* was not significantly different with or without IPTG (Table 4) but was reduced by a factor of ∼5 relative to that in *mug20^+^* (with or without *ade6-3101*). By contrast, the Mug20-LacI fusion promoted *ade6 – arg1* recombination at wild-type frequency and *ade-3101* recombination at enhanced level but was insensitive to IPTG (Supplementary Tables 4B and S6). Thus, in Mug20-LacI, Mug20 has enhanced activity at *ade6-3101* and retains wild-type activity elsewhere, but LacI has lost IPTG-sensitivity although it apparently retains *lacO* binding activity. In LacI-Mug20, Mug20 has enhanced activity at *ade6-3101* but has reduced activity elsewhere, and LacI apparently binds *lacO* with IPTG-sensitivity. These unexpected phenotypes presumably reflect complex interactions between fused Mug20 and LacI, which may in turn reflect complex interactions among the native LinE proteins. A similar, unexpected phenotype is also reported in mice: the Gal4-BD-Spo11 fusion has reduced DSB frequency at many endogenous DSB hotspots, although it creates DSB hotspots at other sites devoid of detectable DSBs in wild type (56).

**Table 4.** IPTG differentially inactivates LacI fusions with Mug20

Ade^+^ per 10^6^ spores					*ade6 – arg1* (cM)			
Allele x *ade6-52*	No fusion (*mug20^+^*)		LacI-Mug20 (*mug20-252*)		No fusion (*mug20^+^*)		LacI-Mug20 (*mug20-252*)	
**(A)**	− IPTG	+ IPTG	− IPTG	+ IPTG	− IPTG	+ IPTG	− IPTG	+ IPTG
*ade6-M375*	369 ± 26	272 ± 44	23 ± 7	43 ± 18	76 ± 14	71 ± 22	16 ± 3	16 ± 7
*ade6-3101*	311 ± 66	272 ± 64	821 ± 93	94 ± 18	71 ± 17	46 ± 10	14 ± 4	14 ± 4
	No fusion (*mug20^+^*)		Mug20-LacI (*mug20-231*)		No fusion (*mug20^+^*)		Mug20-LacI (*mug20-231*)	
**(B)**	− IPTG	+ IPTG	− IPTG	+ IPTG	− IPTG	+ IPTG	− IPTG	+ IPTG
*ade6-M375*	250, 310	240, 260	350, 390	360, 180	71 ± 17	80 ± 21	86 ± 24	57 ± 12
*ade6-3101*	190, 230	300, 210	1400, 1400	1600, 1200	64 ± 14	64 ± 14	76 ± 19	71 ± 17

Intragenic (Ade^+^ per 10^6^ viable spores) and intergenic (cM) recombinant frequencies were assayed in either the absence (*mug20^+^*) or presence of the LacI-Mug20 (*mug20-252*) or Mug20-LacI (*mug20-231*) fusion and with or without IPTG (10 mM). Data for *ade6* intragenic recombination are mean ± SEM from five crosses in (**A**); data from crosses on two separate days are shown for crosses in (**B**). Data for *ade6 – arg1* intergenic recombination are from pooled (homogeneous) data from crosses on separate days; SD is estimated from observed fractions of recombinants. See Supplementary Table S6 for additional data with other Mug20-LacI fusions.

### Recombination at the *ade6-3101* hotspot is similar to that in wild type

To determine the other molecular requirements for recombination at and near the novel *ade6-3101* hotspot, we tested the requirement for other proteins involved in DSB formation – the cohesin subunits (Rec8 and Rec11), the other LinE proteins (Rec10, Rec25 and Rec27), the DSB-forming protein (Rec12; Spo11 homolog), and one of its essential partners (Mde2) (6,35,41,57,58). These tests used both LacI-Mug20 and Mug20-LacI. As expected, both intra- and inter-genic recombination at or near the *ade6-3101* hotspot were essentially completely dependent on Rec10, Rec12 and Mde2 (Table 5), as they are in wild-type (*mug20^+^*) strains. Absence of Rec8 and Rec11 reduced recombination by factors of 3–10 for both LacI-Mug20 and Mug20-LacI; for *mug20^+^* the reductions were much greater, by factors ≥100. Interestingly, both Rec25 and Rec27 were also partially required with LacI-Mug20 (reductions by factors of ∼2–10 in their absence) but much more stringently required with Mug20-LacI and Mug20^+^ (reductions by factors of ∼25–100 in their absence) (Table 5) (35,49). As noted above (Tables 3 and 4), this difference in phenotype suggests that placement of the LacI protein on Mug20 affects function of the LinE complex, possibly through Mug20’s interaction with Rec25-Rec27. Nevertheless, the entire LinE protein complex can function at this hotspot, as at wild-type hotspots (6,34,35).

**Table 5.** *ade6-3101* hotspot recombination requires Rec proteins required for wild-type recombination

LacI-Mug20			Mug20-LacI		Mug20^a^	
*rec* gene deletion	*ade6-3101* x *ade6-52* (intragenic)	*ade6 – arg1* (intergenic)	*ade6-3101* x *ade6-52* (intragenic)	*ade6 – arg1* (intergenic)	*ade6-M26* x *ade6-52* (intragenic)	*ade6 – arg1* (intergenic)
+	518 ± 54 (5)	15 ± 4 (3)	718 ± 39 (8)	49	3800 ± 700	73
*rec8Δ*	115 ± 16 (5)	1.3 (2)	103 ± 18 (4)	<5.9	5 ± 0.3	0.8
*rec11Δ*	201 ± 10 (5)	3.6 (2)	ND^b^	ND	7 ± 1.7	0.7
*rec10Δ*	2.5 ± 1.7 (4)	<2 (2)	5.0 ± 2.0 (4)	<2.4	<8	<0.4
*rec12Δ*	2.4 ± 0.9 (5)	<3 (2)	3.9 ± 1.7 (4)	<2	<5	0.2
*rec25Δ*	233 ± 49 (5)	<3 (2)	ND	ND	34 ± 1.6	3.3 ± 0.4
*rec27Δ*	112 ± 6 (5)	1.4 (2)	11.9 ± 1.8 (4)	1	39 ± 2	2.9 ± 0.6
*mde2Δ*	ND	ND	5.8 ± 1.2 (4)	<2	31^c^	0.4^d^

Intragenic (Ade^+^ per 10^6^ spores) and intergenic (cM) recombinant frequencies were assayed with the LacI-Mug20 fusion (*mug20-252*), the Mug20-LacI fusion (*mug20-231*), or wild-type Mug20 in the presence and absence of the indicated *rec* genes involved in meiotic recombination. Data are mean ± SEM from (n) crosses. For crosses with either zero or one recombinant, recombinant frequency was calculated at the upper 95% confidence interval based on the Poisson distribution.

^a^Data for *rec25Δ* and *rec27Δ* are from (49), and *mde2* data are from (58); other data are from (41) except for *ade6 – arg1* in *rec12Δ* from (82).

^b^ND, not determined.

^c^Data are for *ade6-M26* x *ade6-469*. *mde2^+^* gave 8850 Ade^+^/10^6^ viable spores.

^d^Data are for *leu2-120* x *lys7-2*. *mde2^+^* gave 14.6 cM.

### Double-strand breaks are strongly induced by Mug20-LacI around the *ade6-3101 lacO* array

**Figure 2.**
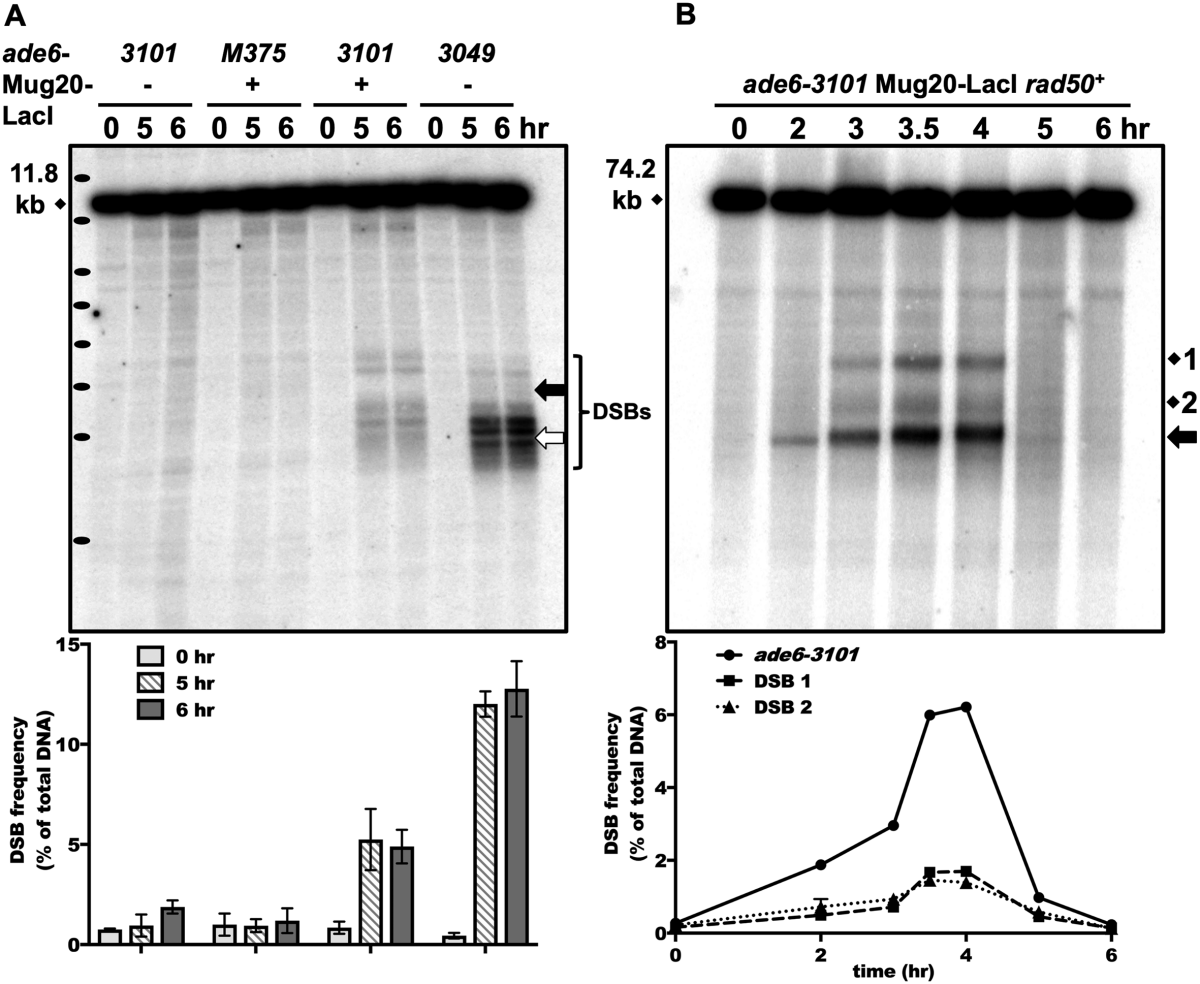
Linear element fusion protein Mug20-LacI induces abundant DSBs at the *ade6-3101* hotspot. (**A**) Formation and accumulation of DSBs in *rad50S* strains with *ade6-3101* containing 8 *lacO* operators (at the thick black arrow) alone, with Mug20-LacI alone (*mug20-231*), or with both to generate the *ade6-3101* hotspot. *ade6-3049* (thick white arrow) is a non-LacI-*lacO* DSB hotspot control. Bracket indicates the ∼2 kb region of DSB formation in the two strains. Cells were induced for meiosis and harvested at the indicated times. DNA was digested with *Bsr*GI and analyzed by electrophoresis and Southern blot hybridization using a probe at the right end of the 11.8 kb fragment with *ade6*. Black ovals on the left margin indicate a DNA ladder (1 kb Plus, Invitrogen; from the top 15, 10, 8, 7, 6, 5, 4, and 3 kb). Quantification is based on 2 or 3 blots from two independent inductions; error bars indicate the range or SEM. (**B***)* Early formation and timely repair of DSBs in a *rad50^+^* strain with *ade6-3101* and Mug20-LacI (*mug20-231*). DNA was analyzed as in (A) after digestion with *Pme*I using a probe at the right end of the 74.2 kb fragment with *ade6*. DSBs at *ade6-3101* are indicated by the thick black arrow (∼20 kb fragment); endogenous DSB sites 1 and 2 are 15 and 5 kb from *ade6*. Quantification is based on two independent inductions; error bars (some invisible) indicate the range. Note that DSBs at *ade6-3101* are visible before replication is complete at 3 h, but DSBs at endogenous site 1, site 2, and *mbs1* are not (see also Supplementary Figure S1).

We tested the induction of meiotic DSBs at the *lacO* hotspot in the presence of either *ade6-3101* or the Mug20-LacI fusion protein alone or both together. We observed distinct, meiosis–specific DSBs only when both *ade6-3101* and Mug20-LacI were present (Figure 2A), as expected from recombination enhancement requiring both elements (Tables 2 and 4). As at wild-type DSB hotspots, DSBs were maximal at 4 h after induction of meiosis in *rad50^+^* and accumulated as expected in the *rad50S* (K81I) mutant, in which DSBs are repaired very slowly (52) (Figure 2). In a *rad50^+^* strain DSBs at *ade6-3101* (Figure 2B, arrow) were detectable as early as 2 h after induction, when DNA replication was beginning (Supplementary Figure S2A). In contrast, DSBs at nearby endogenous DSB sites 1 and 2 and *mbs1* on another chromosome (Figure 2B and Supplementary Figure S2B) appeared only after replication was completed at 3 h, as previously observed (52,55). To test if these early DSBs were formed before replication, we tested an *mde2Δ* strain, in which endogenous DSBs and meiotic recombination are severely reduced (58), and the expression of Mde2 is blocked when replication is blocked (42), showing that DSBs are dependent on replication. Recombination at *ade6-3101* with Mug20-LacI was very strongly reduced, to the same level as in *rec10Δ* and *rec12Δ* (Table 5). This demonstrates that the hotspot is dependent on Mde2 and the early DSBs observed must be formed immediately after replication. This result suggests that Mug20-LacI can bind to the *ade6-3101 lacO* array before and independent of normal (wt) LinE loading (see Discussion, Implications for the mechanism of DSB hotspot competition and interference). All DSBs were repaired about the same time as in wild type (*mug20*^+^). On a short (11.8 kb) *Bsr*GI fragment, we observed clear bands corresponding to DSBs flanking a DSB-free region at the *lacO* array in *ade6-3101* (Figure 2A). This indicates that the Mug20-LacI fusion binds to the *lacO* array and induces breaks to both sides flanking the array. The DSB hotspot allele *ade6-3049*, analyzed for comparison, also showed DSBs flanking the binding site of its hotspot-determinant, the transcription factor Atf-Pcr1 (55).

### DSBs induced by Mug20-LacI have negligible competition with neighboring DSB hotspots but retain DSB interference

**Figure 3.**
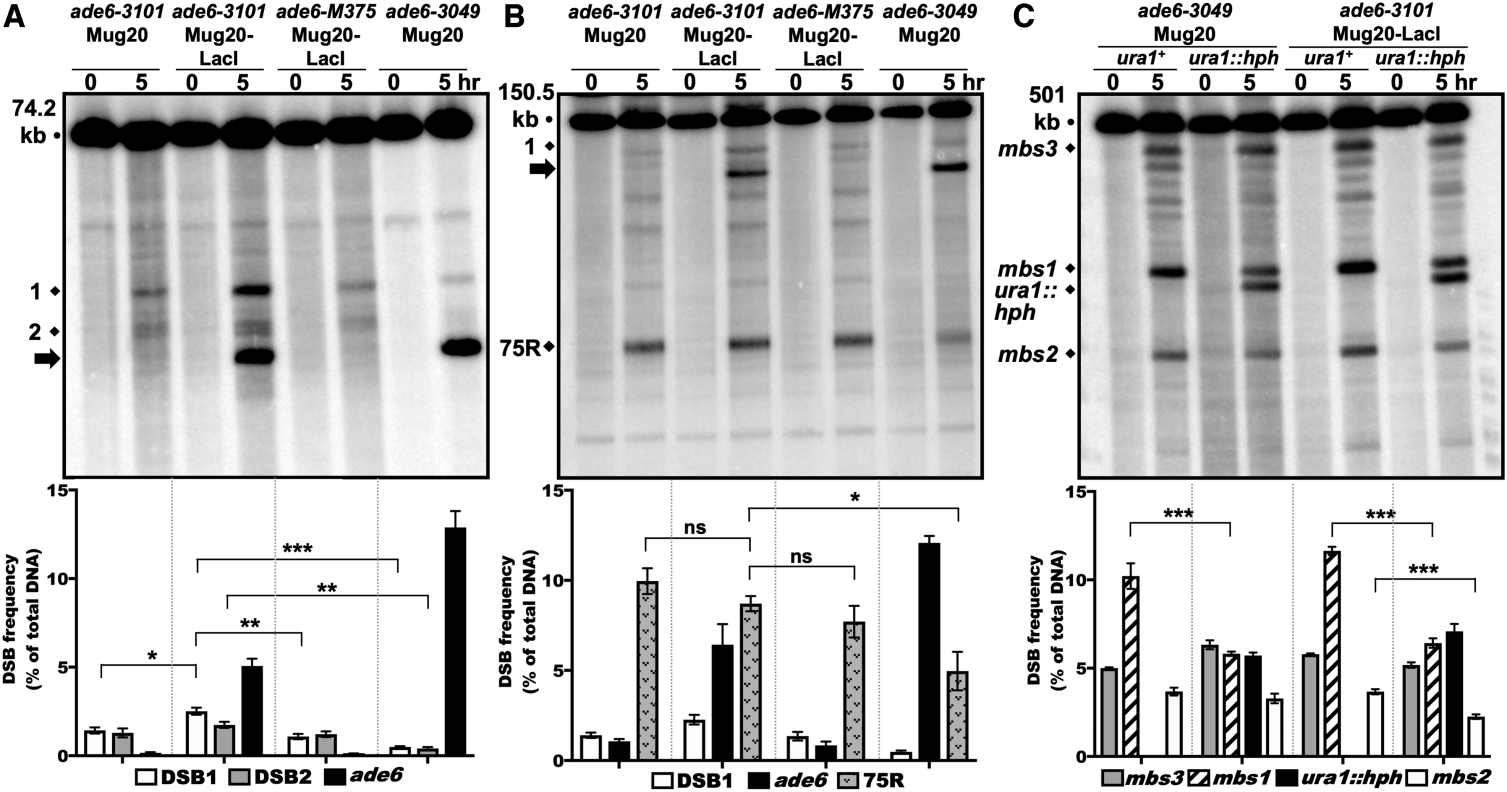
The Mug20-LacI fusion protein lacks DSB hotspot competition at the *ade6-3101* hotspot but retains competition at the endogenous *mbs1* hotspot. *rad50S* strains were induced for meiosis, and DNA analyzed as in Figure 2. In each panel, quantification is based on n blots from two independent inductions; error bars indicate SEM (range for *n* = 2). (**A**) *ade6-3049* but not *ade6-3101* plus Mug20-LacI (*mug20-231*) competes with close hotspots; *ade6-M375* is a non-hotspot control. DNA was digested with *Pme*I and analyzed on three to five blots with a probe at the right end of the 74.2 kb fragment containing *ade6*. DSBs at sites 1 and 2 were significantly less frequent with *ade6-3049* than with *ade6-M375* (***P < 0.0001 for site 1 and **P = 0.0004 for site 2) but were not less frequent with the *ade6-3101* hotspot (thick black arrow, ∼20 kb). Rather, DSBs at site 1 were moderately stimulated by *ade6-3101* plus Mug20-LacI (*mug20-231*) versus *ade6-3101* alone or *M375*: *P = 0.011 or **0.004, respectively). DSBs at site 2 were not significantly different (*P* = 0.18 or 0.054, respectively). (**B**) *ade6-3049* but not the *ade6-3101* hotspot (DSBs indicated by thick black arrow, ∼110 kb) competes with a distant hotspot. DNA was digested with *Sac*II and analyzed on three or four blots with a probe at the right end of the 150.5 kb fragment with *ade6*. Only *ade6-3049* competed with the strong DSB hotspot 75 kb to the right of *ade6* (75R) (*P = 0.015) or with weaker DSBs in between. (**C**) Mug20-LacI manifests DSB competition on another chromosome. DNA was digested with *Not*I and analyzed on four to seven blots with a probe at the left end of the 501 kb *Not*I fragment J. *mbs1* was competed by the artificial hotspot *ura1::hph* in both Mug20 and Mug20-LacI strains (***P < 0.0001). *mbs2*, an endogenous hotspot 100 kb to the left of *mbs1*, was also competed by *ura1::hph* in Mug20-LacI strains (***P < 0.0001). DSBs at *mbs3*, 200 kb to the right of *mbs1*, did not differ significantly in these strains. Strains with no black bar (first and third from the left) are *ura1^+^*.

In cells with wild-type LinEs, DSBs show both competition and interference (19). Competition is the reduction of DSB frequency upon introduction of a nearby hotspot (or increase upon deletion of one hotspot of a pair). Interference is the occurrence of two nearby DSBs on one DNA molecule less frequently than the product of the individual DSB frequencies (as expected from DSB independence). These features act locally, over ∼200 kb regions. We tested these features at the *ade6-3101* hotspot with Mug20-LacI. Remarkably, we observed three distinct bands corresponding to weak endogenous DSB hotspots about 15 and 5 kb from *ade6*, whether *ade6-3101* and Mug20-LacI were present or not (Figure 3A, marked by 1 and 2, a doublet). A strong DSB hotspot ∼75 kb to the right of *ade6* and weaker DSB hotspots in the intervening interval also were not competed by the *ade6-3101* hotspot (75R, Figure 3B). These DSBs were, however, competed by *ade6-3049* as previously observed (19), indicating that the lack of DSB competition is a special feature of the *ade6-3101* hotspot. Interestingly, not only does DSB competition appear lost, but the endogenous DSB hotspot 15 kb away (marked by 1) was stimulated in the presence of Mug20-LacI and *ade6-3101*. DSBs at site 1 were ∼2-fold more frequent in this strain than in strains with only Mug20-LacI or *ade6-3101* (1.43% and 1.08%, respectively, in the single mutants and 2.5% in the double mutant; *P* = 0.01 and 0.0004, respectively); the more distant 75 kb DSB was not detectably stimulated (Figure 3A and B).

We did, however, observe weak DSB competition between *ade6-3101* and a DSB hotspot created by substitution of the *tel1* ORF with the *natMX6* drug-resistance determinant. Insertion of a drug-resistance determinant often forms a strong DSB hotspot, and these hotspots compete and interfere with endogenous hotspots (19). The DSBs at *ade6-3101* were reduced from 5.42% to 3.56% – a reduction of 1/3 – when the *tel1::natMX6* hotspot was introduced (Supplementary Figure S3A). This reduction was, however, markedly less than that observed at *ade6-3049* (4-fold reduction) or at the 75R DSB (2-fold reduction; Supplementary Figure S3A). The DSB hotspot created by the *tel1::natMX6* substitution may have different properties than the other endogenous DSBs nearby.

To test whether the Mug20-LacI fusion protein was functionally defective for DSB competition, perhaps by interfering with LinE complex assembly or activity, we investigated DSB hotspot competition at the *mbs1* hotspot on chromosome I (*ade6* is on chromosome 3). DSB hotspots - including *mbs1* - are dependent on the LinE proteins (6,41). A substitution of the hygromycin-resistance determinant (*hphMX6*) in *ura1* 15–20 kb from the strong endogenous hotspot *mbs1* created a strong DSB hotspot that competed with *mbs1*, reducing its DSB frequency by a factor of 1.8 (from 10.2 to 5.8%; Figure 3C). The same factor of reduction (1.8; from 11.6 to 6.3%) occurred in a strain with the Mug20-LacI fusion (Figure 3C), indicating that the lack of competition at *ade6-3101* is not due to lack of competitive activity by the Mug20-LacI fusion. We infer that DSB competition depends on the manner of LinE protein loading onto the DSB hotspot sites (see Discussion, Implications for the mechanism of DSB hotspot competition and interference).

**Figure 4.**
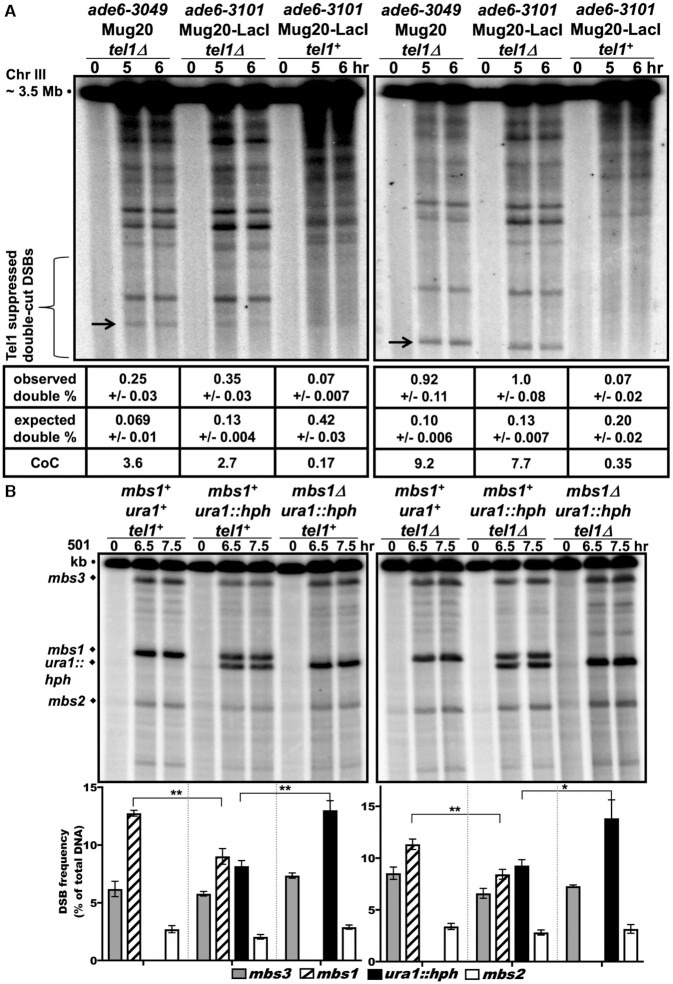
The *ade6-3101* hotspot manifests Tel1-dependent DSB interference; Tel1-independent DSB competition indicates separate mechanisms. *rad50S* strains were induced for meiosis, and DNA analyzed as in Figure 3, except meiosis was at 25°C in (B). (**A**) Both *ade6-3049* and *ade6-3101* manifest Tel1-dependent DSB interference. DNA was digested with NotI and analyzed on four to six blots with a probe between *ade6* and the 75R DSB hotspot (left panel) or between *ade6* and the *tel1*L hotspot near *tel1* (right panel). Double-cut DSBs (black arrows; 75 kb left and 40 kb right) were evident in *tel1Δ* (left and middle lane sets) but not in *tel1^+^* (right lane set). Coefficients of coincidence (CoC; mean ± SEM) show positive DSB interference (1 - CoC) in *tel1^+^* and negative interference in *tel1Δ*. Single-cut DSBs and frequencies are visible in Supplementary Figure S3 using a different radioactive probe. (**B**) DSB competition at *mbs1* is Tel1-independent. DNA was digested with *Not*I and analyzed on three to seven blots with a probe at the left end of the 501 kb *Not*I fragment J. DSBs at both *mbs1^+^* and *ura1::hph* hotspots were reduced in the presence of the other hotspot (compare the double hotspot in the middle lane set to either single hotspot; **P = 0.007 for *mbs1^+^* and **P = 0.0034 for *ura1::hph*), indicating mutual DSB competition. This competition was also present without Tel1 (right panel; **P = 0.002 for *mbs1^+^* and *P = 0.018 for *ura1::hph*).

We next assayed DSB interference between *ade6-3101* and an endogenous DSB hotspot ∼75 kb away (Figure 4A). DSB interference requires the DNA damage-response protein kinase Tel1 (ATM homolog) (19,24). The double-cut fragment was readily seen in a *tel1Δ* strain with *mug20*^+^ and the *ade6-3049* hotspot, as well as in a *tel1Δ* strain with Mug20-LacI and the *ade6-3101* DSB hotspot. It was much more frequent than expected from independence, as measured by the coefficient of coincidence (CoC; the frequency of observed double-cut DNA divided by the product of the frequency of each single-cut DNA) (Supplementary Figure S3A and B). A CoC < 1 indicates positive interference (I = 1 - CoC). The CoC was 3.6 in *mug20*^+^*ade6-3049 tel1Δ* and 2.7 in Mug20-LacI *ade6-3101 tel1Δ*. In contrast, very little double-cut fragment was detected in the isogenic *tel1^+^* strain with Mug20-LacI and *ade6-3101* (Figure 4A, left blot; CoC = 0.17). Similar results were observed between a*de6* and a DSB hotspot 40 kb to the opposite side, near *tel1*. Here, the CoC was 9.2 in *mug20*^+^*ade6-3049 tel1Δ* and 7.7 in Mug20-LacI *ade6-3101 tel1Δ*. Again, very little double-cut fragment was detected in the isogenic *tel1^+^* strain with Mug20-LacI and *ade6-3101* (Figure 4A, right blot; CoC = 0.35). These data agree with previous data of endogenous DSB hotspot pairs (19) and indicate Tel1-dependent DSB interference with Mug20-LacI and the *ade6-3101* hotspot.

In *S. cerevisiae*, DSB interference but not competition depends on Tel1 (24,25); we thus measured DSB competition in a *tel1Δ* strain at *mbs1*, as assays of competition at *ade6* are complicated by its proximity to the *tel1* locus. In both *tel1^+^* and *tel1Δ* strains, we observed mutual competition between *mbs1* and *ura1::hph—*the DSB frequency of each hotspot was reduced in the presence of the other (Figure 4B). The results of these experiments at 25°C were similar to those at 34°C (Figure 3C), indicating temperature-independence of DSB competition. These observations confirm that DSB competition is Tel1-independent and agree with DSB competition and interference being separable at *ade6-3101* with Mug20-LacI. They are consistent with DSB competition arising during the loading of LinE proteins at DSB hotspots before DSB formation and DSB interference arising by action of Tel1 after the first DSB has been made (25,28) (see Discussion, Implications for the mechanism of DSB hotspot competition and interference).

### Recombination intermediates at the *ade6-3101* hotspot are similar to those at the stronger *ade6-3049* DSB hotspot but show less frequent interhomolog DSB repair

Our analyses showed a discrepancy between the DSB and recombinant frequencies when comparing the *ade6-3101* hotspot and the Atf1-Pcr1-dependent hotspots *ade6-M26* and *ade6-3049* (Figure 1). DSBs at the *ade6-3101* hotspot (Figures 2 and 3A) were 4-fold more frequent than those at the *ade6-M26* hotspot (55). However, the recombinant frequency with *ade6-3101* was 5 times less frequent than that with *ade6-M26*: in crosses with *ade6-52*, *ade6-3101* produces at most 1400 Ade^+^/10^6^ viable spores (Tables 1–5), but *ade6-M26* produces ∼4000 Ade^+^/10^6^ viable spores (59). Thus, the recombinant:DSB ratio is ∼20-fold lower for *ade6-3101* than for *ade6-M26*, even though they are at the same place in the *ade6* gene (Figure 1). One possible explanation for this discrepancy is frequent repair of DSBs at the *ade6-3101* hotspot without formation of joint molecules between the broken and intact homolog. An alternative is frequent DSB repair with the sister chromatid, which cannot yield recombinants.

**Figure 5.**
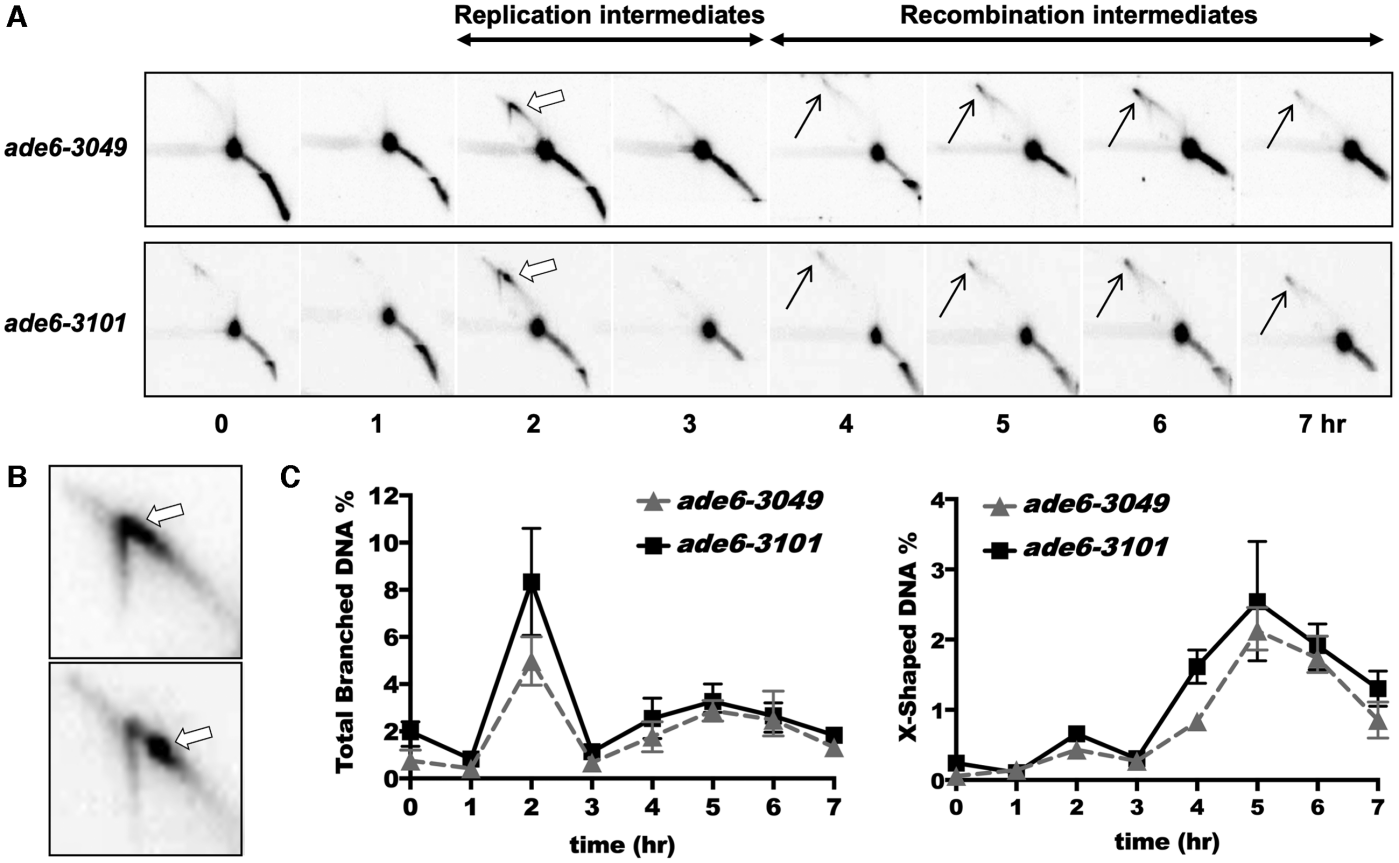
Joint DNA molecules arise at similar time and frequency at the *ade6-3101* and *ade6-3049* DSB hotspots. *mus81Δ* strains were induced for meiosis, and DNA, digested with *BsrG*I, was analyzed by two-dimensional gel electrophoresis and Southern blot hybridization using a probe near the right end of the 11.8 kb fragment with *ade6*. (**A**) Branched DNA molecules, predominantly replication intermediates (Y-shaped; thick white arrows), arose at 2 h, and recombination intermediates (X-shaped Holliday junctions; thin black arrows) appeared at 4–6 h. The prominent spot is the parental DNA fragment. (**B**) Expanded view of replication arc at 2 h, showing a prominent pause or DSB site in the *ade6-3101* strain (bottom panel) but not in the *ade6-3049* strain (upper panel). (**C**) For quantification, branched DNA (structures above the linear DNA arc) was normalized to total DNA. Quantification is based on two or three blots from two independent inductions; error bars indicate the range or SEM.

To examine these possibilities, we determined the total amount of homologous recombination intermediates (X-shaped joint molecules, black arrows in Figure 5) generated at each hotspot locus during meiosis. Two hours after induction of meiosis, we observed at the two loci similar levels of replication intermediates (Y-shaped branched molecules, white arrows), which disappeared by 3 h (Figure 5A). One distinct difference was a prominent spot on the Y- arc with the *ade6-3101* hotspot (Figure 5B). Since DSBs were initiated as replication was beginning (Figure 2B and Supplementary Figure S1A), replication may pause at *ade6-3101* bound by Mug20-LacI; this view suggests that loading, but not DSB formation, precedes replication, as noted above.

This spot was transient, and meiotic progression was not impeded. Recombination intermediates started to appear at 4 h and accumulated until 7 h, due to absence of the Mus81-Eme1 Holliday junction (HJ)-resolving factor in the *mus81Δ* strain used (60) (Figure 5A). Remarkably, the frequency of the X-shaped recombination intermediates was similar at both *ade6-3101* and *ade6-3049* (Figure 5C), even though the DSB frequency differed by a factor of 2.5 (Figures 2 and 3A). The total (X- plus Y-shaped) intermediates were also similar in frequency at both loci (Figure 5C). This result shows that homologous recombination intermediates (HJs) were readily formed at *ade6-3101* but leaves unexplained its low recombinant frequency.

**Figure 6.**
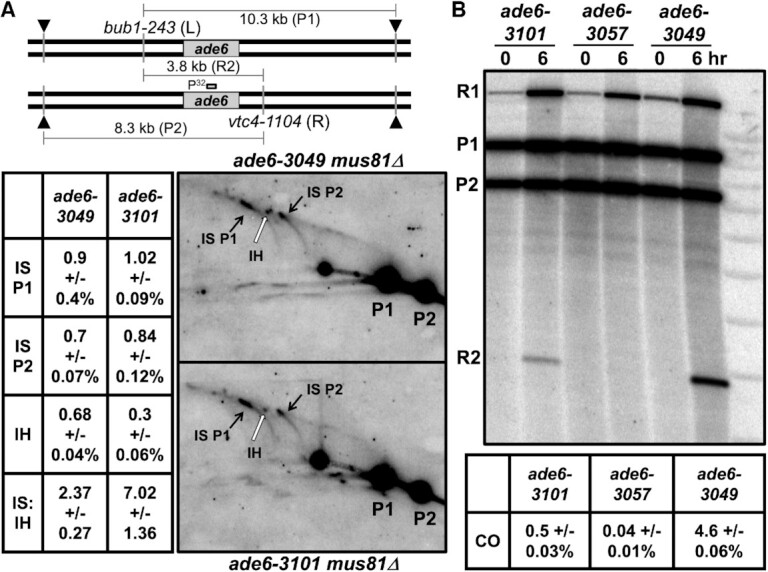
Less interhomolog DSB repair occurs at the *ade6-3101* hotspot than at the *ade6-3049* hotspot, and crossover DNA is strongly reduced.Diploid strains heterozygous for *bub1-243* (L) and *vtc4-1104* (R), flanking *ade6* were used to distinguish intersister (IS) and interhomolog (IH) Holliday junctions (HJs) in (A) and crossover DNA in (B) (53). (**A**) *mus81Δ* strains were induced for meiosis and harvested at 5 h. DNA was digested with *Pml*I and *Sca*I and analyzed as in Figure 5 using a probe near the middle of *ade6*. IS HJs (black arrows) and IH HJs (white arrows) were quantified from three (*ade6-3049*) or six (*ade6-3101 mug20-231*) blots from two independent inductions; data are IS L/P1, IS R/P2, and IH/[(P1 + P2)/2], each as % ± SEM, where P1 and P2 are parental DNAs 1 and 2, respectively. The IS to IH ratio of HJs is indicated. (**B**) Strains with the indicated homozygous *ade6* alleles were induced for meiosis and harvested at 5 hr; DNA was analyzed as in (A), except electrophoresis was in only one dimension. *ade6-3057* is a non-hotspot control (Figure 1). R1 and R2 are reciprocal recombinant fragments. The fraction of crossover fragment, 2 × R2/(P1 + P2) because R1 can also arise from a partial restriction digestion, is based on three to five blots from two independent meiotic inductions; error bars indicate the SEM.

DSB repair can occur by joint molecule formation with the homolog, which can generate a genetic recombinant, or with the sister chromatid, which cannot. Preferential repair with the sister could explain the low recombinant frequency with the *ade6-3101* hotspot. We thus compared the ratio of intersister to interhomolog (IS:IH) X-shaped recombination intermediates (single HJs) at these hotspots. Heterozygous restriction sites flanking the hotspots allowed IS *vs*. IH distinction (53) (Figure 6A). For *ade6-3049*, the IS:IH ratio was 2.3, close to that reported previously (53). For the *ade6-3101* hotspot with Mug20-LacI the IS:IH ratio was 6.5, or 3 times higher than that with *ade6-3049*. The total HJ frequency was nearly the same (2.3% and 2.2%) for each hotspot, as noted in the previous experiments (Figure 5). Thus, preferential repair of DSBs at the *ade6-3101* hotspot with the sister can account for some but not all of the difference in recombinant frequency (see Discussion, Alterations in partner choice for DSB repair).

### Physical assay of crossover DNA also reveals low recombinant frequency at *ade6-3101* hotspot

It remained possible that the low recombinant frequency with the *ade6-3101* hotspot in genetic assays (Tables 1–5) reflects incomplete recovery of recombinants, *e.g*., inviability of spores after DSB formation and repair at the *ade6-3101* hotspot. Alternatively, the heterologous sequence created by the *lacO* substitution might impede strand invasion and recombinant formation. (Note that the *lacO* substitution was homozygous in the physical HJ assays above but heterozygous in the genetic recombination assays.) To test these possibilities, we assayed before spore formation total recombinant DNA with a physical assay employing the heterozygous restriction sites flanking *ade6* used in Figure 6A in a strain homozygous for *ade6-3101* (*i.e*., no large heterology present). Recombinant DNA bearing both restriction cut-sites was assayed by gel electrophoresis and Southern blot hybridization (Figure 6B). The *ade6-3101* with Mug20-LacI produced 0.50% recombinant DNA fragment, 9 times less than *ade6-3049* produced (4.6%); the non-hotspot control *ade6-3057* (nine bp from *ade6-3049*) produced even less (0.04%). Thus, these physical assays of recombinants parallel the genetic assays (Tables 1–5). Below, we discuss possible explanations of these seemingly disparate data for recombination intermediates (DSBs and HJs) and final recombinants (genetic and physical).

## DISCUSSION

The results presented here show that localization of meiotic linear element (LinE) proteins to a chromosomal site is sufficient to generate a DSB and recombination hotspot, complementing the necessity of LinE proteins reported previously (6). Here, we discuss these results, which provide further evidence for the DSB hotspot-clustering model previously proposed to aid solving two long-standing problems in meiosis – determining the molecular mechanisms of DSB hotspot competition and DSB and crossover interference (19).

### Fusion proteins lead to DSB hotspots

In earlier, related research, *S. cerevisiae* Spo11 protein, with the active site for DSB formation, was localized to a chromosomal site by fusing Spo11 to the DNA-binding domain (BD) of the Gal4 transcription activator protein; this fusion led to a DSB and recombination hotspot at a Gal4-binding site in the *GAL2* promoter and multiple other DSB hotspots (23,27,61). Fusion of Gal4-BD to any of seven proteins in the Spo11 complex also leads to new DSB hotspots at *GAL2* (62). Fusion of Spo11 to Gal4-BD stimulates DSB formation at >200 sites in mice (56), and fusion of Spo11 to other DNA site-specific binding proteins, such as Cas9–sgRNA and zinc fingers, results in novel DSB and crossover hotspots in *S. cerevisiae* (63). In an alternative approach, introduction of the DNA sequence for binding of each of three transcription factors into the *S. pombe ade6* gene results in recombination hotspots dependent on the respective endogenous (unfused) transcription factor (10,64). Similar to previous work in *S. cerevisiae* that tethered Ssp1, a subunit of the histone-methylating COMPASS complex, to Gal4-BD to create DSB hotspots (65,66), we have created hotspots using the LinE proteins that determine endogenous meiotic hotspots and act before DSB formation. Thus, proteins in addition to Spo11 and its partners can be used for genetic engineering of meiotic recombination (67).

DSBs are not formed precisely at the localization site; rather, DSBs occur to the sides of the site, spread over as much as ∼1 kb regions to both sides in *S. pombe*. This is true for the *ade6-3101* hotspot described here (Figure 2A) and for the Atf1-Pcr1-dependent hotspots *ade6-M26*, *ade6-3049* and other *ade6* alleles (55) (Figure 2A). We infer that the LinE complex binds to special chromosomal sites and then directs the Rec12-complex to cut the DNA to either side but not where the localization factors (LinE proteins or Atf1-Pcr1) are bound. This result and the strict Rec10- and Rec12-dependence of recombination (Table 5) suggest that, although the LinE-LacI fusion protein is loaded onto its bound hotspot site differently, subsequent DSB formation proceeds in a manner similar to that in wild type.

### Implications for the mechanism of DSB hotspot competition and interference

The introduced DSB hotspots in *S. cerevisiae* mentioned above all compete with endogenous DSB hotspots, a distinct difference from the *ade6-3101* DSB hotspot studied here. In *S. cerevisiae* hotspot competition is, however, not always observed (68,69). The *ade6-3101* hotspot also differs from the endogenous *S. pombe* hotspots examined to date, which manifest both hotspot competition and DSB interference (19). In *S. pombe*, introduction of a hotspot such as *ade6-3049* without any fusion protein reduces DSB or recombination frequency at nearby hotspots (within ∼200 kb) (Figure 3) (19,70). When introduced with the Mug20-LacI fusion protein, the *ade6-3101* DSB hotspot, however, did not compete with endogenous DSB hotspots on either side of the *lacO* array, even as close as 5 kb or as far as 75 kb (Figure 3). DSBs at the *ade6-3101* hotspot do, however, manifest Tel1-dependent DSB interference, just like DSBs at endogenous hotspots (Figure 4) (19). As in *S. cerevisiae* (25), DSB competition is largely independent of Tel1 (Figure 4). These observations confirm that DSB competition and DSB interference are separable phenomena and invite discussion of their molecular mechanisms.

**Figure 7.**
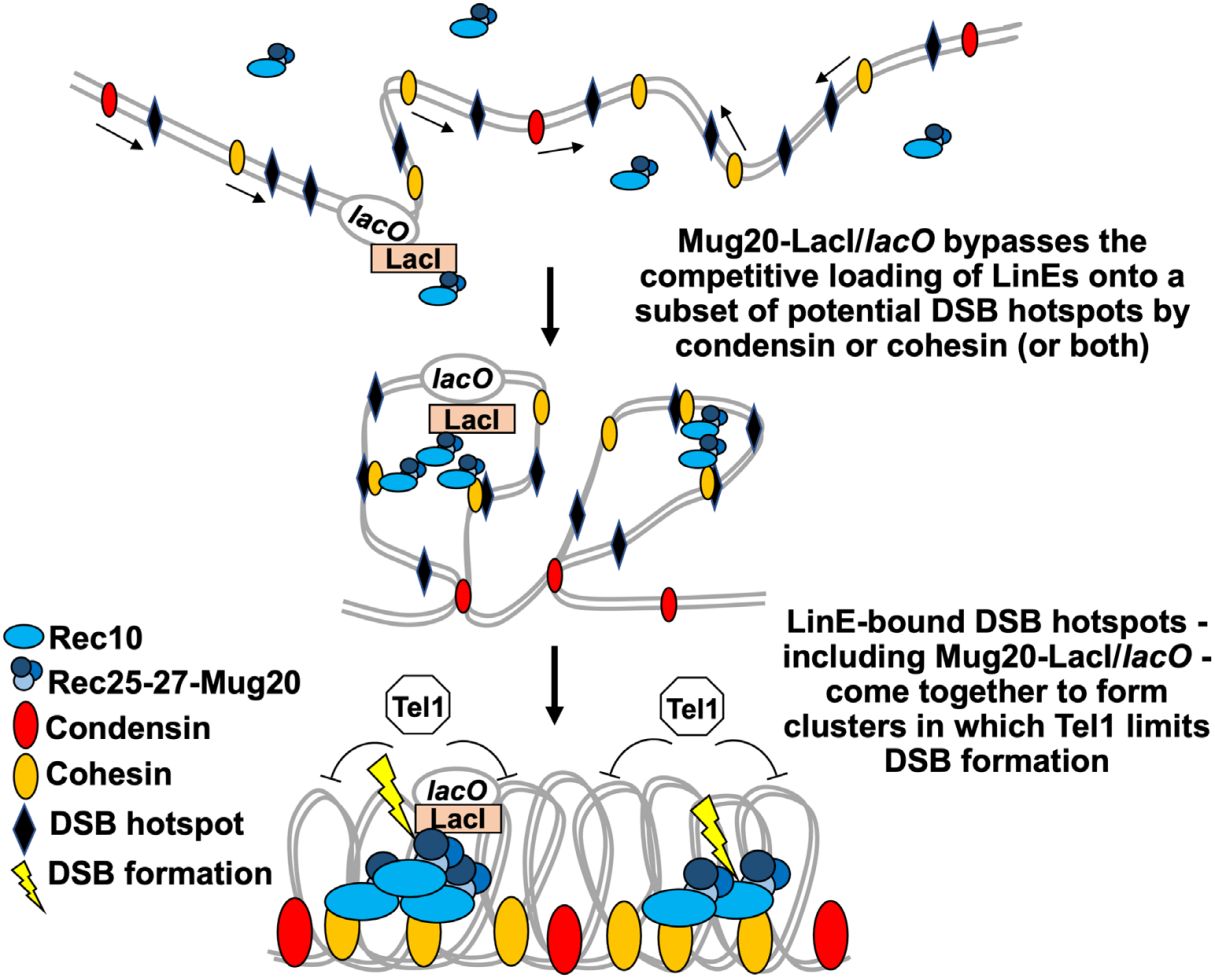
Model for DSB competition arising from the competitive loading of LinE complexes onto DSB hotspots. A loader, such as cohesin or condensin, moves along paired sister chromatids (thin black arrows) and loads LinE complexes (blue circles and ovals) onto a limited number of potential DSB hotspot sites. This prevents other sites in this traversed interval from being bound by LinEs and therefore limits DSBs to only the LinE-loaded sites (DSB competition). The *ade6-3101* hotspot with a *lacO* array allows independent loading of Mug20-LacI and thus lacks DSB competition. The loader (cohesin or condensin) groups the LinE-hotspot complexes, including the Mug20-LacI-bound site, into a cluster, in which a DSB is formed. This DSB activates Tel1 protein kinase to prevent further DSB formation in that cluster (DSB interference).

We have proposed that DSB hotspot competition and DSB interference reflect the clustering of a limited number of DSB hotspots, perhaps only two, over chromosomal regions up to ∼200 kb and the formation of a limited number of DSBs, perhaps only one, in each cluster (19). The absence of competition by the *ade6-3101* hotspot suggests that competition occurs during loading of the hotspot-determinant proteins (LinEs), preceding DSB formation as previously proposed (20,23,26,27). We suggest that some factor, such as cohesin or condensin, loads LinEs onto sites with DSB hotspot potential. As the factor moves along the chromosome, it loads additional LinEs onto subsequently encountered potential hotspots on that chromosome or the sister chromatid (*i.e*., in *cis*) and clusters these LinE-bound hotspots together (Figure 7). At some point, the moving factor ceases loading, limiting potential hotspots, which manifests as competition of the DSB sites. Loading likely does not occur at all potential hotspots; some have greater potential for loading than others, thus accounting for the variation in DSB hotspot strength (6,7). Introduction of a strong potential hotspot would reduce the frequency of loading at sites subsequently encountered by the loader; deletion of a hotspot would have the opposite effect, thus accounting for localized hotspot competition in *cis* (19). Cohesin and condensin separately form topologically associated domains (TADs) over ∼80 kb and ∼300 kb, respectively, in *S. pombe* mitotic cells (71), similar to the distance (∼200 kb) over which competition and interference occur in *S. pombe* (19). In support of this view, the hotspot at *ade6-3101* with Mug20-LacI is much less dependent on cohesin subunits Rec8 and Rec11 than are endogenous hotspots (Table 5), suggesting that cohesin's role in loading of LinEs has been at least partially bypassed; condensin has yet to be tested.

This model may have general features that apply to other species. Cohesin is necessary for proper DSB regulation in many species (43,72–75). Studies of meiotic chromosome organization in mammals and *S. cerevisiae* have revealed similar dynamic compaction and loop extrusion (76–79). In *S. cerevisiae*, this meiotic chromosome structure depends on the cohesin subunit Rec8, and chromosome compaction also depends on proteins of the synaptonemal complex (SC) (77). Interestingly, in mammals TADs are diminished during meiosis (76,78,79), a change dependent on the SC (79). These data show that meiotic chromosome structure is unique and dynamic, and that loading of recombination factors and bringing these factors together through changes in chromosomal domains may be a conserved feature in meiotic recombination. They also highlight differences, and further investigation of the structure of *S. pombe* meiotic chromosomes will be of interest.

In this scenario, a hotspot at which the LinE was artificially loaded, such as by the Mug20-LacI fusion protein at *ade6-3101*, would not be loaded by the normal loader (*e.g*., cohesin or condensin) and would not compete with neighboring hotspots (Figure 7). Alternative scenarios, such as self-loading of DSB-promoting proteins limited by localized diffusion and self- enhanced binding (28), are also possible. In these alternative scenarios, however, it is not clear how DSB competition occurs only in *cis*. In either loading scenario, once the proteins are localized at potential DSB hotspot sites, the neighboring LinE proteins may assemble as condensates to form a higher-order regulatory cluster, as observed for proteins of the *S. cerevisiae* DSB-forming complex (28).

DSB interference is proposed to arise from the formation of a limited number of DSBs in a cluster, *i.e.*, after formation of the hotspot cluster (19). Once one DSB is formed in a cluster, some factor, such as the Tel1 DNA damage-response protein kinase, is activated and prevents further DSB formation; Tel1 is required for DSB and crossover interference (19,24,80). Once the LinE complex is loaded onto the hotspot, even by its own action, the *ade6-3101* hotspot could enter the surrounding cluster and be subject to Tel1’s limitation of DSB formation. This scenario accounts for the Tel1-dependent DSB interference observed with the *ade6-3101* hotspot (Figure 4). These observations support the clustering model (19) and encourage further investigation of the mechanism of LinE loading and a search for a loader, such as cohesin or condensin.

### Alterations in partner choice for DSB repair

While the combination of LinE-LacI and *lacO* array clearly created both a DSB hotspot and a recombination hotspot, we were surprised that the DSB frequency was so high (Figures 2–4), given the modest frequency of recombinants at the *lacO* site in *ade6-3101* (Tables 1–5). This discrepancy between DSB and recombinant frequencies may have multiple sources. One source may be the large heterology imparted by the *lacO* array: recombinant frequencies in the absence of a LinE-LacI fusion protein steadily decreased as the length of heterology increased (Table 2). But the *lacO* array was homozygous (hence, no heterology) in the physical recombinant assays, which showed ∼10 times fewer recombinants with *ade6-3101* than with *ade6-3049* (Figure 6B). But only some of this reduction is due to the 2-fold lower DSBs at *ade6-3101* than at *ade6-3049* (Figures 2 and 3). Interhomolog Holliday junctions (HJs), which unlike intersister HJs can be converted into recombinants, also were 2-fold less abundant with *ade6-3101* than with *ade6-3049* (Figure 6A), but these factors still do not fully account for the paucity of recombinants. In addition to their established role in DSB formation (6), LinEs may have a role in directing DSB repair, as proposed from LinE structures evolving from early to late meiosis (31,36,40). In particular, the manner of loading LinEs at hotspots, self-loading *vs*. cohesin- or condensin-mediated loading for example, may influence both partner choice for HJ-formation and crossover *vs*. non-crossover preference during DSB repair. The 3-fold higher IS:IH ratio of HJs with Mug20-LacI than with *ade6-3049* (Figure 6A) may be related to the previously observed crossover invariance – more uniform crossover frequency than DSB frequency (53). The mechanism for invariance has been unknown, but DSBs at hotspots with high IS:IH ratio are more dependent on the histone variant H2A.Z than are DSBs in cold regions or at weak hotspots (18,81). It was proposed that H2A.Z promotes LinE binding to chromatin-bound cohesin; this view suggests that crossover invariance is directly related to LinE function and loading.

The LinE-LacI–*lacO* DSB and recombination hotspots studied here support the hotspot-clustering model for DSB competition and interference. They also reveal new features of meiotic DSB hotspots and their activating proteins. Additional investigations of LinE-LacI–*lacO* hotspots should help understand further the molecular mechanism of meiotic recombination.

## Supplementary Material

gkab1253_Supplemental_File

## Notes

Present address: Joshua D. Cho, Dept. of Pathology, Columbia University, New York, NY 10032, USA.

Present address: Mridula Nambiar, Department of Biology, Indian Institute of Science Education and Research, Pune 411008, India.
