# Supplementary material for: Redirecting meiotic DNA break hotspot determinant proteins alters localized spatial control of DNA break formation and repair
Source: Nucleic Acids Res. 2021 Dec 30;50(2):899–914. doi: 10.1093/nar/gkab1253 (PMC8789058; doi:10.1093/nar/gkab1253)
Supplement: gkab1253_Supplemental_File [file gkab1253_supplemental_file.pdf]

Supplementary Data

Redirecting meiotic DNA break hotspot determinant proteins alters localized spatial controls of DNA break formation and repair

Randy W. Hyppa, Joshua D. Cho, Mridula Nambiar, and Gerald R. Smith

Table S1. *S. pombe* strains

Strain	Genotype	Used in
GP13	*h- ade6-52*	Table 2
GP935	*h- ade6-52 ura4-D182*	Tables 2, 4A, and 4B
GP5275	*h- ade6-3049 pat1-114 mus81::kanMX6*	Figure 5
GP5366	*h+ ade6-3049 rad50S pat1-114*	Figures 2A, 3A and B
GP6104	*h+ ade6-3095::ura4*$^{+}$ *ura4-D18*	Making *ade6::lacO*
GP6656	*h-/h- ade6-3049/ade6-3049* (+ *vtc4-1104*)/(*bub1-243* +) *pat1-114/pat1-114* (*lys3-37* +)/(+ *ura1-61*) *mbs1-24/mbs1-25* (*his4-239* +)/(+ *lys4-95*)	Figure 6B
GP6657	*h-/h- ade6-3049/ade6-3049* (+ *vtc4-1104*)/(*bub1-243* +) *pat1-114/pat1-114* (*lys3-37* +)/(+ *ura1-61*) *mbs1-24/mbs1-25 mus81::kanMX6/ mus81::kanMX6* (*his4-239* +)/(+ *lys4-95*)	Figure 6A
GP6713	*h-/h- ade6-3057/ade6-3057* (+ *vtc4-1104*)/(*bub1-243* +) *pat1-114/pat1-114* (*lys3-37* +)/(+ *ura1-61) mbs1-24/mbs1-25* (*his4-239* +)/(+ *lys4-95*)	Figure 6B
GP7301	*h- ade6-52 ura4-D18 rec10-260::ura4+*	Making *rec10* fusions
GP8210	*h- ade6-M26 ura4-D18 arg1-14 rec25-256::ura4+*	Making *rec25* fusions
GP8211	*h- ade6-M26 ura4-D18 arg1-14 mug20-258::ura4+*	Making *mug20* fusions
GP8513	*h- ade6-M26 ura4-D18 arg1-14 rec27-257::ura4+*	Making *rec27* fusions
GP8795	*h- ade6-52 ura4-D18 arg1-14 rec25-256::ura4+*	Making *rec25* fusions
GP8796	*h- ade6-52 ura4-D18 arg1-14 mug20-258::ura4+*	Making *mug20* fusions
GP8797	*h- ade6-52 ura4-D18 arg1-14 rec27-257::ura4+*	Making *rec27* fusions
GP8799	*h- ade6-52 ura4-D18 arg1-14 mug20-231:lacI*	Table 2
GP8801	*h- ade6-52 ura4-D18 rec10-233:lacI*	Table 1
GP8846	*h- ade6-52 ura4-D18 rec27-232:lacI*	Table 1
GP8848	*h+ ade6-3098 ura4-D18 arg1-14*	Table 2
GP8849	*h- ade6-3099 ura4-D18 arg1-14*	Table 2
GP8859	*h+ ade6-52 ura4-D18*	Tables 1, 2, and 3
GP8871	*h+ ade6-3101 ura4-D18*	Table 2
GP8873	*h+ ade6-3098 ura4-D18 arg1-14 mug20-231:lacI*	Table 2

GP8874	*h+ ade6-3099 ura4-D18 arg1-14 mug20-231:lacI*	Table 2
GP8875	*h- ade6-52 ura4-D18 mug20-231:lacI*	Tables 1, 2, 3 and 4B
GP8897	*h+ ade6-3103::ura4*$^{+}$ *bub1-243 ura4-D18*	Making *ade6::lacO*
GP8898	*h+ ade6-3103::ura4*$^{+}$ *vtc4-1104 ura4-D18*	Making *ade6::lacO*
GP8899	*h+ ade6-3101 ura4-D18 rec10-233:lacI*	Table 1
GP8900	*h+ ade6-3101 ura4-D18 arg1-14*	Tables 4A and 4B
GP8901	*h- ade6-3101 ura4-D18 arg1-14*	Tables 1, 2, and 3
GP8902	*h+ ade6-3101 ura4-D18 arg1-14 mug20-231:lacI*	Tables 1, 2, 3, and 4B
GP8904	*h+ ade6-3101 ura4-D18 arg1-14 rec27-232:lacI*	Table 1
GP8976	*h+ ade6-3101 ura4-D18 arg1-14 rec25-230:lacI*	Table 1
GP8985	*h- tel1::kanMX6*	Making *tel1::natMX6*
GP8995	*h+ ade6-3101 pat1-114 rad50S*	Figures 2A, 3A and B
GP8999	*h+ ade6-3102 ura4-D18 arg1-14*	Table 2
GP9002	*h+ ade6-3102 ura4-D18 arg1-14 mug20-231:lacI*	Table 2
GP9064	*h+ ade6-3106 ura4-D18 arg1-14*	Table 2
GP9065	*h+ ade6-3107 ura4-D18 arg1-14*	Table 2
GP9066	*h+ ade6-3106 ura4-D18 arg1-14 mug20-231:lacI*	Table 2
GP9067	*h+ ade6-3107 ura4-D18 arg1-14 mug20-231:lacI*	Table 2
GP9072	*h+ ade6-3111 ura4-D18 arg1-14 mug20-231:lacI*	Table 2
GP9073	*h+ ade6-3111 ura4-D18 arg1-14*	Table 2
GP9074	*h+ ade6-3108 ura4-D18 arg1-14 mug20-231:lacI*	Table 2
GP9075	*h+ ade6-3108 ura4-D18 arg1-14*	Table 2
GP9076	*h+ ade6-3109 ura4-D18 arg1-14 mug20-231:lacI*	Table 2
GP9077	*h+ ade6-3109 ura4-D18 arg1-14*	Table 2
GP9078	*h- ade6-52 ura4-D18 mug20-252:lacI*	Tables 3, 4A, and 5
GP9080	*h- ade6-52 ura4-D18 mug20-254:lacI*	Table 3
GP9083	*h+ ade6-3101 ura4-D18 arg1-14 mug20-254:lacI*	Table 3
GP9099	*h+ ade6-M375 ura4-D18 arg1-14 mug20-231:lacI*	Tables 2 and 4B
GP9102	*h- ade6-3101 pat1-114 rad50S mug20-231:lacI*	Figures 2A, 3A and B
GP9125	*h- ade6-52 ura4-D18 mug20-252:lacI rec11::kanMX*	Table 5
GP9128	*h+ ade6-M375 ura4-D18 arg1-14*	Tables 2, 4A, and 4B
GP9152	*h- ade6-52 ura4-D18 mug20-252:lacI rec10-175::kanMX*	Table 5
GP9153	*h- ade6-52 ura4-D18 mug20-252:lacI rec12-169::kanMX*	Table 5
GP9154	*h- ade6-52 ura4-D18 mug20-252:lacI rec27-184::kanMX*	Table 5

GP9155	*h- ade6-52 ura4-D18 mug20-252:lacI rec25-180::kanMX*	Table 5
GP9156	*h- ade6-52 ura4-D18 rec25-230:lacI*	Table 1
GP9157	*h+ ade6-3101 ura4-D18 arg1-14 mug20-252:lacI*	Tables 3, 4A, and 5
GP9189	*h+ ade6-3101 ura4-D18 arg1-14 mug20-252:lacI rec10-175::kanMX*	Table 5
GP9190	*h+ ade6-3101 ura4-D18 arg1-14 mug20-252:lacI rec12-169::kanMX*	Table 5
GP9191	*h+ ade6-3101 ura4-D18 arg1-14 mug20-252:lacI rec27-184::kanMX*	Table 5
GP9192	*h- ade6-M375 pat1-114 rad50S mug20-231:lacI*	Figures 2A, 3A and B
GP9193	*h+ ade6-3101 ura4-D18 arg1-14 mug20-252:lacI rec11::kanMX*	Table 5
GP9201	*h+ ade6-3101 ura4-D18 arg1-14 mug20-252:lacI rec25-180::kanMX*	Table 5
GP9254	*h+ ade6-3101 ura4-D18 arg1-14 mug20-252:lacI rec8-176::kanMX*	Table 5
GP9267	*h- ade6-52 ura4-D18 mug20-252:lacI rec8-176::kanMX6*	Table 5
GP9389	*h- ade6-3101 mug20-231 pat1-114 mus81::kanMX6*	Figure 5
GP9501	*h+ ade6-M375 ura4-D18 arg1-14 mug20-252:lacI*	Table 4
GP9582	*h-/h- ade6-3101/ade6-3101* (+ *vtc4-1104*)/(*bub1-243* +) *mug20-231/mug20-231 pat1-114/pat1-114 mus81::kanMX6/mus81::kanMX6* (*his4-239* +)/(*lys4-95* +)	Figure 6A
GP9673	*h-/h- ade6-3101/ade6-3101* (+ *vtc4-1104*)/(*bub1-243* +) *mug20-231/mug20-231 pat1-114/ pat1-114* (*his4-239* +)/(*lys4-95* +)	Figures 2B, 6B, and S2
GP9901	*h- ade6-3049 pat1-as.L95G:kanMX6 rad50S*	Figures 3C and 4B; making *ura1::hphMX6*
GR15	*h- ade6-3049 pat1-as.L95G:kanMX6 rad50S tel1::natMX6 ura4-aim*	Figures 4A, 4B, and S3
GR77	*h- ade6-3049 pat1-as.L95G:kanMX6 rad50S ura1::hphMX6*	Figures 3C and 4B
GR105	*h+ ade6-3049 pat1-as.L95G:kanMX6 rad50S mbs1-20 ura1::hphMX6*	Figure 4B
GR119	*h- ade6-3049 pat1-as.L95G:kanMX6 rad50S ura1::hphMX6 tel1::natMX6*	Figure 4B
GR134	*h- ade6-3031 pat1-as.L95G:kanMX6 rad50S mug20-231:lacI ura1::hphMX6*	Figure 3C
GR135	*h+ ade6-3031 pat1-as.L95G:kanMX6 rad50S mug20-231:lacI*	Figures 3C, 4A, and S3
GR137	*h- ade6-3031 pat1-as.L95G:kanMX6 rad50S mug20-231:lacI tel1::natMX6*	Figures 4A and S3
GR141	*h+ ade6-52 pat1-as.L95G:kanMX6 rad50S mbs1-20 ura1::hphMX6 tel1::natMX6*	Figure 4B
GR255	*h- ade6-52 ura4-D18 mug20-231:lacI rec8-176::kanMX6*	Table 5

GR256	*h+ ade6-3101 ura4-D18 arg1-14 mug20-231:lacI rec8-176::kanMX6*	Table 5
GR257	*h- ade6-52 ura4-D18 mug20-231:lacI rec10-175::kanMX*	Table 5
GR258	*h+ ade6-3101 ura4-D18 arg1-14 mug20-231:lacI rec10-175::kanMX*	Table 5
GR259	*h- ade6-52 ura4-D18 mug20-231:lacI rec12-169::kanMX*	Table 5
GR260	*h+ ade6-3101 ura4-D18 arg1-14 mug20-231:lacI rec12-169::kanMX*	Table 5
GR261	*h- ade6-52 ura4-D18 mug20-231:lacI rec27-184::kanMX*	Table 5
GR262	*h+ ade6-3101 ura4-D18 arg1-14 mug20-231:lacI rec27-184::kanMX*	Table 5
GR263	*h- ade6-52 ura4-D18 mug20-231:lacI mde2::hphMX6*	Table 5
GR264	*h+ ade6-3101 ura4-D18 arg1-14 mug20-231:lacI mde2::hphMX6*	Table 5

Strains were constructed by standard matings (Smith, 2009) or as described in Materials and Methods. Genealogies are available upon request. "*::*" indicates a substitution (deletion and insertion); "*:*" indicates an insertion without a deletion. Sources of alleles other than mating-type and standard auxotrophies are: *ade6-3095::ura4*$^+$ (Hyppa and Smith, 2010); *bub1-243* (Hyppa and Smith, 2010); *mbs1-20* (Fowler et al., 2018); *mbs1-24 and mbs1-25* (Cromie et al., 2006); *mug20-258::ura4*$^+$ (Ma et al., 2017); *mus81::kanMX6* (Boddy et al., 2001); *pat1-114* (Iino and Yamamoto, 1985); *pat1-as.L95G:kanMX6* (Guerra-Moreno et al., 2012); *rad50S* (Farah et al., 2002); *rec8-176::kanMX6* (Farah et al., 2005); *rec10-175::kanMX6* (Ellermeier and Smith, 2005); *rec10-260::ura4*$^+$ (Ma et al., 2017); *rec11::kanMX6* (Kitajima et al., 2003); *rec12-169::kanMX6* (Davis and Smith, 2003); *rec12-171::ura4*$^+$ (Davis and Smith, 2003); *rec25-180::kanMX6* (Martin-Castellanos et al., 2005); *rec25-256::ura4*$^+$ (Ma et al., 2017); *rec27-184::kanMX6* (Martin-Castellanos et al., 2005); *rec27-257::ura4*$^+$ (Ma et al., 2017); *tel1::kanMX6* (Y. Yamada, O. Limbo, P. Russell)*; vtc4-1104* (Hyppa and Smith, 2010).

Table S2. Oligonucleotides

OL #	Sequence (5’ → 3’)
3530	GCC GTC ATT TTA GAT GAA TTC AAG
3531	AGC ATC TTT CAT CTT GCT TAA ATC
3532	GCG GAT AAC AAT TGT GCA CGT TAT TAA CAA CTG
3533	TCA CAA TTG CTA GTG GGT CAG TAA ATG ATG CAT
3534	AGATCCTGAACCACTTCCAGAACCATTACCAGTAGATTGAAAACAC
3536	CTG GAA GTG GTT CAG GAT CTG TGA AAC CAG TAA CGT TAT ACG
3537	GAG TTT CAA TCG TAA TTT AGC TTA TTT ACT TGT CAT CGT CAT CTT T
3539	ATA AGC TAA ATT ACG ATT GAA ACT CAA AGC TAA ATT TAT TCC AAC TCT
3542	CAC AAT TGC TAG TTG GTT GCT GCA ATG ACA C
3543	AGC GGA TAA CAA TTC ACC AGC AAT AAT CAC AC
3547	AGA TCC TGA ACC ACT TCC AGA ACC AAA ATT GTC GAG AAT AGC TTT ATG
3548	ATT TCA GAG ATC TAA TGT CAT ACT G
3549	GTA TGA CAT TAG ATC TCT GAA ATT TAC TTG TCA TCG TCA TCT T
3550	AGA TCC TGA ACC ACT TCC AGA ACC TAC TTC TGT ATT TGT TGG TTG
3551	AGT CAT TTT AAA CAC ATT ATA TAA ACT AG
3552	ATA ATG TGT TTA AAA TGA CTT TAC TTG TCA TCG TCA TCT TTA TAA TC
3553	AGA TCC TGA ACC ACT TCC AGA ACC CTT AAA CAT CAA ACT GTC
3554	CTT GAA CCT ATA GTA TCA AAT TTA TTG
3555	AAT TTG ATA CTA TAG GTT CAA GTT ACT TGT CAT CGT CAT CTT
3576	CCA TCG CTT AAA CAT CAT CCA CTG TGT GCC GA
3577	TGA TGT TTA AGC GAT GG
3579	GCT CAA TTT CAG TTG TTA ATA AGT TAA AAT AGT GAA GGA ACT AGT
3580	TTT AAG ATT GCA CTT CCT G
3581	GCA AAA TTC AGG AAG TGC AAT CTT AAA GTT AAA ATA GTG AAG GAA CTA GT
3587	TGA TGT TTA AGC GAT GGG
3588	TTA TTA ACA ACT GAA ATT GAG C
3592	GTG CTT CCT TTT ACA GGA AC
3594	TGT AAA AGG AAG CAC TCC ACT GTG TGC CGA
3596	TTT CAA CCC TCC CTT TTG
3605	TAG ATC CTG AAC CAC TTC C
3608	TAA ATT TCA GAG ATC TAA TGT CAT AC
3610	GAA GTG GTT CAG GAT CTA TGA AAC CAG TAA CGT TAT ACG ATG TCG
3611	CAT TAG ATC TCT GAA ATT TAC TGC CCG CTT TCC AGT CG
3613	GTT GCG TGT GAT TAT TGC TCC ACT GTG TGC CGA
3614	CAG CCT TTT CTA AAA CAA TCT CTT TAT GTT AAA ATA GTG AAG GAA CTA GT
3615	GCA ATA ATC ACA CGC AAC
3616	AAA GAG ATT GTT TTA GAA AAG G
3617	CCA AAA GGG AGG GTT GAA AAT GTT AAA ATA GTG AAG GAA CTA GT
3618	TGT TGT GGT TCT TGA TAA CAT G
3619	GTT ATC AAG AAC CAC AAC ATG AAA CCA GTA ACG TTA TAC GAT GTC G
3620	GGT TCT GGA AGT GGT TCA GGA TCT ATG TCT GAA ATT ATA ACG TCT TTG
3621	AGA TCC TGA ACC ACT TCC AGA ACC CTG CCC GCT TTC CAG TCG
4416	AAG CAT CAG GAT TGA TAC GG
4417	ATG ATT GCTT AGC ACC TTC C
4418	GAG ACC AAC CAC TCC TTA GAA GCA ATA TCT GGG AAT CGC TAG TCT TAA GCC TCA AAT CTG TGG AAT TGT TGA TAT AAG CGC GGA TCC CCG GGT TAA TTA A
4419	GGG CTG TTT GAC CAC CAA AGG TAA CAT ATA TCG AAT CGG GGC GCT CTT GTT TGA TCA CCT TAC GAA CAA AGT CAG CAT TAG AAT TCG AGC TCG TTT AAA C
4461	ACC ATA CTA CTA AGA CAA CAA CCA AAG TGA ATG GGT AAT TCA AGA CTA AAA GAA AAG AGG ATC ACG GAA ATT AAT TAA AAC GGA TCC CCG GGT TAA TTA A
4462	AAC TCT TAC CGA TAA TTC AGC TAC CTG CTT ACC TAC AGT ATC TAT CAT TGA ATT AAA AAG GGG AAA TTC TGA ATT ATT AAG AAT TCG AGC TCG TTT AAA C

Table S3. Progenitor plasmids		
Plasmid (pseudonym)	Description	Source or reference
pRH6	pUC19 + 4.4 kb *Sal*I fragment containing *bub1-243* and *ade6-3057*	(Hyppa and Smith, 2010)
pJC1	*bub1-243 ade6*$^+$	NEB Q5 site-directed mutagenesis using pRH6 and oligos OL3530 and OL3531.
pUC-TALO3 (pLO3)	pUC19 + 3x *lacO*	(Unnikrishnan et al., 2012)
pUC-TALO8 (pLO8)	pUC19 + 8x *lacO*	(Unnikrishnan et al., 2012)
pRS406-CMV-LacI-NLS-3FLAG (pRS)	pRS406 + *lacI-NLS-3FLAG*	(Unnikrishnan et al., 2012)
pLM06	pFY20 + 3.2 kb *Xma*I fragment containing *rec25*$^+$	(Ma et al., 2017)
pLM08	pFY20 + 1.4 kb *Kpn*I fragment containing *rec27*$^+$	(Ma et al., 2017)
pLM09	pFY20 + 1.5 kb *Kas*I fragment containing *mug20*$^+$	(Ma et al., 2017)
pYL150	pSP2 + 5.6 kb *Pvu*II fragment containing *rec10*$^+$	(Lin and Smith, 1995)
PCR2.1-hph	pCR2.1 + hygromycin B resistance gene	(Sato et al. 2005)

Table S4. *ade6* and LinE mutant alleles generated for this study

			Source[1]	
Allele (pseudonym)	Description[2]	Plasmid product[3]	PCR1 (Plasmid, OL#s)	PCR2 (Plasmid, OL#s)
ade6-3098 (L1)	1x*lacO* sub at *ade6* nt 171-197	pJC2	pJC1 (3532, 3533)	NA[4]
ade6-3099 (R1)	1x*lacO* sub at *ade6* nt 1343-1369	pJC3	pJC1 (3542, 3543)	NA
ade6-3101 (L8)	8x*lacO* sub at *ade6* nt 86-339	pJC12	pJC1 (3577, 3580)	pLO8 (3576, 3581)
ade6-3102 (L3)	3x*lacO* sub at *ade6* nt 86-204	pJC13	pJC1 (3587, 3588)	pLO3 (3576, 3579)
ade6-3106 (L3, R3)	*ade6-3102* + *ade6-3110*	pJC30	pJC13 (3592, 3596)	pLO3 (3594, 3617)
ade6-3107 (L3, R8)	*ade6-3102* + *ade*6-*3111*	pJC31	pJC13 (3615, 3616)	pLO8 (3613, 3614)
ade6-3108 (L8, R3)	*ade6-3101* + *ade6-3110*	pJC28	pJC12 (3592, 3596)	pLO3 (3594, 3617)
ade6-3109 (L8, R8)[5]	*ade6-3101* + *ade6-3111*	pJC29	pJC12 (3615, 3616)	pLO8 (3613, 3614)
ade6-3110 (R3)	3x*lacO* sub at *ade6* nt 1425-1545	pJC17	pJC1 (3592, 3596)	pLO3 (3594, 3617)
ade6-3111 (R8)	8x*lacO* sub at *ade6* nt 1338-1593	pJC16	pJC1 (3615, 3616)	pLO8 (3613, 3614)
mug20-231 (Mug20-LacI)	*mug20*-(GS)$_4$-*lacI-NLS-3FLAG*	pJC7	pLM09 (3547, 3548)	pRS (3549, 3536)
mug20-252 (LacI-Mug20)	*lacI*-(GS)$_4$-*mug20*	pJC22	pLM09 (3618, 3620)	pRS (3619, 3621)
mug20-254	*mug20*-(GS)$_4$-*lacI*	pJC27	pJC7 (3605, 3608)	pRS (3610, 3611)
rec10-233 (Rec10-LacI)	*rec10*-(GS)$_4$-*lacI-NLS-3FLAG*	pJC11	pYL150 (3553, 3554)	pRS (3536, 3555)
rec25-230 (Rec25-LacI)	*rec25*-(GS)$_4$-*lacI-NLS-3FLAG*	pJC5	pLM06 (3534, 3539)	pRS (3536, 3537)
rec27-232 (Rec27-LacI)	*rec27*-(GS)$_4$-*lacI-NLS-3FLAG*	pJC9	pLM08 (3550, 3551)	pRS (3536, 3552)

[1] Plasmids and alleles with two sources (PCR1 and PCR2) were created by combining, with the NEB Gibson Assembly kit, the PCR products from the indicated plasmid and oligos. Those with only one source (*ade6-3098* and *ade6-3099*) were created with the NEB Q5 Site-Directed Mutagenesis Kit using the indicated plasmid and oligos.

[2] Product of construction of the indicated allele. Nucleotide numbering starts at the first adenine of the *ade6* open reading frame. NLS (nuclear localization signal) amino acid sequence is PKKKRKV. FLAG amino acid sequence is DYKDDDDK. *lacO* DNA sequence is 5' ACTAGCAATTGTGAGCGGATAACAATT 3'. All *lacO* arrays, other than single substitutions, are flanked by 15 non-*ade6* nucleotides (TCCACTGTGTGCCGA) to their left and 23 or 25 nucleotides (ACTAGTTCCTTCACTATTTTAAC) to their right present in the plasmids pLO3 and pLO8. (GS)$_4$ (linker) amino acid sequence is GSGSGSGS. "sub," substitution; "nt," nucleotide.

[3] pLO8 = pUC-TALO8; pLO3 = pUC-TALO3; pRS = pRS406-CMV-*lacI-nls-3FLAG*. See Table S3.

[4] Not applicable (see footnote 1).

[5] See text below for nucleotide sequences of *ade6*$^{+}$ and *lacO* substitutions (in red).

***ade6*⁺**

ATGAGCGAAAAACAGGTTGTAGGGATCCTTGGAGGTGGTCAATTGGGCCGAATGATGGTAGAGGCA
GCCCATCGCTTAAACATCAAATGCATCATCTTGGATGCAGCAAATTCTCCTGCCAAACAAATTGATGG
AGGACGTGAGCACATTGATGCATCATTTACTGACCCCGATGCAATTGTTGAACTGTCTAAGAAGTGC
ACGTTATTAACAACTGAAATTGAGCATATTAACACTGATGCCTTGGCAGCCGTTACGAAATCTGTTGC
TGTTGAACCCTCTCCTGCAACTCTGCGATGCATTCAAGACAAATATCTTCAAAAACAGCATTTACAGG
TTTTTAAGATTGCACTTCCTGAATTTTGCGATGCACCTGACCAGGAAAGTGTTGAAAAAGCAGGCCAA
GAGTTTGGTTATCCTTTTGTACTGAAAAGTAAAACATTGGCTTACGACGGTCGTGGAAATTACGTTGT
TCATCAACCATCTGAGATTCCTACTGCCATCAAAGCACTTGGTGATCGTCCGCTTTATGTTGAAAAGT
TCGTTCCTTTCTCCATGGAAATTGCAGTGATGGTAGTACGCAGTTTAGACGGAAAAGTTTATGCTTAT
CCTACAACTGAGACCATTCAAAAGGATAATGTTTGTCATTTAGTATATGCCCCTGCTCGTCTTCCCTT
CTCAATTCAACAGCGTGCTCAAACCCTTGCCATGGATGCGGTGCGCACTTTTGAAGGCGCTGGTATA
TATGGTGTAGAGATGTTTGTTTTGCAAGATGGTGAGACCATTTTACTCAACGAAATTGCTCCTCGGCC
TCACAATTCAGGTCACTACACCATTGAAGCTTGCCCAACTTCTCAGTTTGAAGCTCACTTACGGGCCA
TATGTGGTCTTCCTTTCAGCGAAATCAACACCCAACTCTCGACTTCCACAACTCATGCGTTGATGGTA
AATATTTTAGGTACTGATGATCCTGATTATGTTTCAAAGATCGCTAAACGTTCTCTGTCCATTCCCGGT
GCAACTTTGCATCTTTATGGTAAAGCTGAATCTAGAAAGGGTCGCAAGATGGGACACGTTACCATCA
TTTCTGATTCACCTCAAGAATGTGAACGTAGGTATCAGATGCTTCTTGACGTCAAAGATCCTGTCGAA
TCACCTGTTGTTGGTATTATCATGGGTTCGGATTCTGATTTAAGCAAGATGAAAGATGCTGCCGTCAT
TTTAGATGAATTCAAGGTGCCTTACGAACTTACTATTGTTTCAGCTCACCGCACACCAGATCGCATGG
TTACTTATGCTCGTACCGCAGCTTCAAGAGGGTTGCGTGTGATTATTGCTGGTGCTGGTGGTGCCGC
TCATTTGCCTGGTATGGTTGCTGCAATGACACCTCTTCCAGTAATCGGCGTTCCTGTAAAAGGAAGC
ACTCTTGACGGAGTTGACTCTCTTCACTCTATTGTTCAGATGCCTCGAGGTGTCCCTGTCGCCACTG
TTGCTATCAATAATAGCCAAAATGCCGGTATTTTAGCCTGTCGTATACTTGCTACATTTCAACCCTCCC
TTTTGGCTGCTATGGAGAGCTTTATGGACAATATGAAAGAGATTGTTTTAGAAAAGGCTGATAAATTA
GAAAAAGTTGGTTGGAAAAATTATTCTGCATAG

***ade6-3101* (L8)**

ATGAGCGAAAAACAGGTTGTAGGGATCCTTGGAGGTGGTCAATTGGGCCGAATGATGGTAGAGGCA
GCCCATCGCTTAAACATCATCCACTGTGTGCCGAACTAGCAATTGTGAGCGGATAACAATTACTAGC
AATTGTGAGCGGATAACAATTACTAGCAATTGTGAGCGGATAACAATTACTAGCAATTGTGAGCGGAT
AACAATTACTAGCAATTGTGAGCGGATAACAATTACTAGCAATTGTGAGCGGATAACAATTACTAGCA
ATTGTGAGCGGATAACAATTACTAGCAATTGTGAGCGGATAACAATTACTAGTTCCTTCACTATTTTAA
CTTTAAGATTGCACTTCCTGAATTTTGCGATGCACCTGACCAGGAAAGTGTTGAAAAAGCAGGCCAA
GAGTTTGGTTATCCTTTTGTACTGAAAAGTAAAACATTGGCTTACGACGGTCGTGGAAATTACGTTGT
TCATCAACCATCTGAGATTCCTACTGCCATCAAAGCACTTGGTGATCGTCCGCTTTATGTTGAAAAGT
TCGTTCCTTTCTCCATGGAAATTGCAGTGATGGTAGTACGCAGTTTAGACGGAAAAGTTTATGCTTAT
CCTACAACTGAGACCATTCAAAAGGATAATGTTTGTCATTTAGTATATGCCCCTGCTCGTCTTCCCTT
CTCAATTCAACAGCGTGCTCAAACCCTTGCCATGGATGCGGTGCGCACTTTTGAAGGCGCTGGTATA
TATGGTGTAGAGATGTTTGTTTTGCAAGATGGTGAGACCATTTTACTCAACGAAATTGCTCCTCGGCC
TCACAATTCAGGTCACTACACCATTGAAGCTTGCCCAACTTCTCAGTTTGAAGCTCACTTACGGGCCA
TATGTGGTCTTCCTTTCAGCGAAATCAACACCCAACTCTCGACTTCCACAACTCATGCGTTGATGGTA
AATATTTTAGGTACTGATGATCCTGATTATGTTTCAAAGATCGCTAAACGTTCTCTGTCCATTCCCGGT
GCAACTTTGCATCTTTATGGTAAAGCTGAATCTAGAAAGGGTCGCAAGATGGGACACGTTACCATCA
TTTCTGATTCACCTCAAGAATGTGAACGTAGGTATCAGATGCTTCTTGACGTCAAAGATCCTGTCGAA
TCACCTGTTGTTGGTATTATCATGGGTTCGGATTCTGATTTAAGCAAGATGAAAGATGCTGCCGTCAT
TTTAGATGAATTCAAGGTGCCTTACGAACTTACTATTGTTTCAGCTCACCGCACACCAGATCGCATGG
TTACTTATGCTCGTACCGCAGCTTCAAGAGGGTTGCGTGTGATTATTGCTGGTGCTGGTGGTGCCGC
TCATTTGCCTGGTATGGTTGCTGCAATGACACCTCTTCCAGTAATCGGCGTTCCTGTAAAAGGAAGC
ACTCTTGACGGAGTTGACTCTCTTCACTCTATTGTTCAGATGCCTCGAGGTGTCCCTGTCGCCACTG
TTGCTATCAATAATAGCCAAAATGCCGGTATTTTAGCCTGTCGTATACTTGCTACATTTCAACCCTCCC
TTTTGGCTGCTATGGAGAGCTTTATGGACAATATGAAAGAGATTGTTTTAGAAAAGGCTGATAAATTA
GAAAAAGTTGGTTGGAAAAATTATTCTGCATAG

***ade6-3102* (L3)**

ATGAGCGAAAAACAGGTTGTAGGGATCCTTGGAGGTGGTCAATTGGGCCGAATGATGGTAGAGGCA
GCCCATCGCTTAAACATCATCCACTGTGTGCCGAACTAGCAATTGTGAGCGGATAACAATTACTAGC
AATTGTGAGCGGATAACAATTACTAGCAATTGTGAGCGGATAACAATTACTAGTTCCTTCACTATTTTA
ACTTATTAACAACTGAAATTGAGCATATTAACACTGATGCCTTGGCAGCCGTTACGAAATCTGTTGCT

GTTGAACCCTCTCCTGCAACTCTGCGATGCATTCAAGACAAATATCTTCAAAAACAGCATTTACAGGT
TTTTAAGATTGCACTTCCTGAATTTTGCGATGCACCTGACCAGGAAAGTGTTGAAAAAGCAGGCCAA
GAGTTTGGTTATCCTTTTGTACTGAAAAGTAAAACATTGGCTTACGACGGTCGTGGAAATTACGTTGT
TCATCAACCATCTGAGATTCCTACTGCCATCAAAGCACTTGGTGATCGTCCGCTTTATGTTGAAAAGT
TCGTTCCTTTCTCCATGGAAATTGCAGTGATGGTAGTACGCAGTTTAGACGGAAAAGTTTATGCTTAT
CCTACAACTGAGACCATTCAAAAGGATAATGTTTGTCATTTAGTATATGCCCCTGCTCGTCTTCCCTT
CTCAATTCAACAGCGTGCTCAAACCCTTGCCATGGATGCGGTGCGCACTTTTGAAGGCGCTGGTATA
TATGGTGTAGAGATGTTTGTTTTGCAAGATGGTGAGACCATTTTACTCAACGAAATTGCTCCTCGGCC
TCACAATTCAGGTCACTACACCATTGAAGCTTGCCCAACTTCTCAGTTTGAAGCTCACTTACGGGCCA
TATGTGGTCTTCCTTTCAGCGAAATCAACACCCAACTCTCGACTTCCACAACTCATGCGTTGATGGTA
AATATTTTAGGTACTGATGATCCTGATTATGTTTCAAAGATCGCTAAACGTTCTCTGTCCATTCCCGGT
GCAACTTTGCATCTTTATGGTAAAGCTGAATCTAGAAAGGGTCGCAAGATGGGACACGTTACCATCA
TTTCTGATTCACCTCAAGAATGTGAACGTAGGTATCAGATGCTTCTTGACGTCAAAGATCCTGTCGAA
TCACCTGTTGTTGGTATTATCATGGGTTCGGATTCTGATTTAAGCAAGATGAAAGATGCTGCCGTCAT
TTTAGATGAATTCAAGGTGCCTTACGAACTTACTATTGTTTCAGCTCACCGCACACCAGATCGCATGG
TTACTTATGCTCGTACCGCAGCTTCAAGAGGGTTGCGTGTGATTATTGCTGGTGCTGGTGGTGCCGC
TCATTTGCCTGGTATGGTTGCTGCAATGACACCTCTTCCAGTAATCGGCGTTCCTGTAAAAGGAAGC
ACTCTTGACGGAGTTGACTCTCTTCACTCTATTGTTCAGATGCCTCGAGGTGTCCCTGTCGCCACTG
TTGCTATCAATAATAGCCAAAATGCCGGTATTTTAGCCTGTCGTATACTTGCTACATTTCAACCCTCCC
TTTTGGCTGCTATGGAGAGCTTTATGGACAATATGAAAGAGATTGTTTTAGAAAAGGCTGATAAATTA
GAAAAAGTTGGTTGGAAAAATTATTCTGCATAG

***ade6-3098* (L1)**
ATGAGCGAAAAACAGGTTGTAGGGATCCTTGGAGGTGGTCAATTGGGCCGAATGATGGTAGAGGCA
GCCCATCGCTTAAACATCAAATGCATCATCTTGGATGCAGCAAATTCTCCTGCCAAACAAATTGATGG
AGGACGTGAGCACATTGATGCATCATTTACTGACCCACTAGCAATTGTGAGCGGATAACAATTGTGC
ACGTTATTAACAACTGAAATTGAGCATATTAACACTGATGCCTTGGCAGCCGTTACGAAATCTGTTGC
TGTTGAACCCTCTCCTGCAACTCTGCGATGCATTCAAGACAAATATCTTCAAAAACAGCATTTACAGG
TTTTTAAGATTGCACTTCCTGAATTTTGCGATGCACCTGACCAGGAAAGTGTTGAAAAAGCAGGCCAA
GAGTTTGGTTATCCTTTTGTACTGAAAAGTAAAACATTGGCTTACGACGGTCGTGGAAATTACGTTGT
TCATCAACCATCTGAGATTCCTACTGCCATCAAAGCACTTGGTGATCGTCCGCTTTATGTTGAAAAGT
TCGTTCCTTTCTCCATGGAAATTGCAGTGATGGTAGTACGCAGTTTAGACGGAAAAGTTTATGCTTAT
CCTACAACTGAGACCATTCAAAAGGATAATGTTTGTCATTTAGTATATGCCCCTGCTCGTCTTCCCTT
CTCAATTCAACAGCGTGCTCAAACCCTTGCCATGGATGCGGTGCGCACTTTTGAAGGCGCTGGTATA
TATGGTGTAGAGATGTTTGTTTTGCAAGATGGTGAGACCATTTTACTCAACGAAATTGCTCCTCGGCC
TCACAATTCAGGTCACTACACCATTGAAGCTTGCCCAACTTCTCAGTTTGAAGCTCACTTACGGGCCA
TATGTGGTCTTCCTTTCAGCGAAATCAACACCCAACTCTCGACTTCCACAACTCATGCGTTGATGGTA
AATATTTTAGGTACTGATGATCCTGATTATGTTTCAAAGATCGCTAAACGTTCTCTGTCCATTCCCGGT
GCAACTTTGCATCTTTATGGTAAAGCTGAATCTAGAAAGGGTCGCAAGATGGGACACGTTACCATCA
TTTCTGATTCACCTCAAGAATGTGAACGTAGGTATCAGATGCTTCTTGACGTCAAAGATCCTGTCGAA
TCACCTGTTGTTGGTATTATCATGGGTTCGGATTCTGATTTAAGCAAGATGAAAGATGCTGCCGTCAT
TTTAGATGAATTCAAGGTGCCTTACGAACTTACTATTGTTTCAGCTCACCGCACACCAGATCGCATGG
TTACTTATGCTCGTACCGCAGCTTCAAGAGGGTTGCGTGTGATTATTGCTGGTGCTGGTGGTGCCGC
TCATTTGCCTGGTATGGTTGCTGCAATGACACCTCTTCCAGTAATCGGCGTTCCTGTAAAAGGAAGC
ACTCTTGACGGAGTTGACTCTCTTCACTCTATTGTTCAGATGCCTCGAGGTGTCCCTGTCGCCACTG
TTGCTATCAATAATAGCCAAAATGCCGGTATTTTAGCCTGTCGTATACTTGCTACATTTCAACCCTCCC
TTTTGGCTGCTATGGAGAGCTTTATGGACAATATGAAAGAGATTGTTTTAGAAAAGGCTGATAAATTA
GAAAAAGTTGGTTGGAAAAATTATTCTGCATAG

***ade6-3111* (R8)**
ATGAGCGAAAAACAGGTTGTAGGGATCCTTGGAGGTGGTCAATTGGGCCGAATGATGGTAGAGGCA
GCCCATCGCTTAAACATCAAATGCATCATCTTGGATGCAGCAAATTCTCCTGCCAAACAAATTGATGG
AGGACGTGAGCACATTGATGCATCATTTACTGACCCCGATGCAATTGTTGAACTGTCTAAGAAGTGC
ACGTTATTAACAACTGAAATTGAGCATATTAACACTGATGCCTTGGCAGCCGTTACGAAATCTGTTGC
TGTTGAACCCTCTCCTGCAACTCTGCGATGCATTCAAGACAAATATCTTCAAAAACAGCATTTACAGG
TTTTTAAGATTGCACTTCCTGAATTTTGCGATGCACCTGACCAGGAAAGTGTTGAAAAAGCAGGCCAA
GAGTTTGGTTATCCTTTTGTACTGAAAAGTAAAACATTGGCTTACGACGGTCGTGGAAATTACGTTGT
TCATCAACCATCTGAGATTCCTACTGCCATCAAAGCACTTGGTGATCGTCCGCTTTATGTTGAAAAGT
TCGTTCCTTTCTCCATGGAAATTGCAGTGATGGTAGTACGCAGTTTAGACGGAAAAGTTTATGCTTAT

CCTACAACTGAGACCATTCAAAAGGATAATGTTTGTCATTTAGTATATGCCCCTGCTCGTCTTCCCTT
CTCAATTCAACAGCGTGCTCAAACCCTTGCCATGGATGCGGTGCGCACTTTTGAAGGCGCTGGTATA
TATGGTGTAGAGATGTTTGTTTTGCAAGATGGTGAGACCATTTTACTCAACGAAATTGCTCCTCGGCC
TCACAATTCAGGTCACTACACCATTGAAGCTTGCCCAACTTCTCAGTTTGAAGCTCACTTACGGGCCA
TATGTGGTCTTCCTTTCAGCGAAATCAACACCCAACTCTCGACTTCCACAACTCATGCGTTGATGGTA
AATATTTTAGGTACTGATGATCCTGATTATGTTTCAAAGATCGCTAAACGTTCTCTGTCCATTCCCGGT
GCAACTTTGCATCTTTATGGTAAAGCTGAATCTAGAAAGGGTCGCAAGATGGGACACGTTACCATCA
TTTCTGATTCACCTCAAGAATGTGAACGTAGGTATCAGATGCTTCTTGACGTCAAAGATCCTGTCGAA
TCACCTGTTGTTGGTATTATCATGGGTTCGGATTCTGATTTAAGCAAGATGAAAGATGCTGCCGTCAT
TTTAGATGAATTCAAGGTGCCTTACGAACTTACTATTGTTTCAGCTCACCGCACACCAGATCGCATGG
TTACTTATGCTCGTACCGCAGCTTCAAGAGGGTTGCGTGTGATTATTGCTCCACTGTGTGCCGAACT
AGCAATTGTGAGCGGATAACAATTACTAGCAATTGTGAGCGGATAACAATTACTAGCAATTGTGAGC
GGATAACAATTACTAGCAATTGTGAGCGGATAACAATTACTAGCAATTGTGAGCGGATAACAATTACT
AGCAATTGTGAGCGGATAACAATTACTAGCAATTGTGAGCGGATAACAATTACTAGCAATTGTGAGC
GGATAACAATTACTAGTTCCTTCACTATTTTAACATAAAGAGATTGTTTTAGAAAAGGCTGATAAATTA
GAAAAAGTTGGTTGGAAAAATTATTCTGCATAG

***ade6-3110* (R3)**

ATGAGCGAAAAACAGGTTGTAGGGATCCTTGGAGGTGGTCAATTGGGCCGAATGATGGTAGAGGCA
GCCCATCGCTTAAACATCAAATGCATCATCTTGGATGCAGCAAATTCTCCTGCCAAACAAATTGATGG
AGGACGTGAGCACATTGATGCATCATTTACTGACCCCGATGCAATTGTTGAACTGTCTAAGAAGTGC
ACGTTATTAACAACTGAAATTGAGCATATTAACACTGATGCCTTGGCAGCCGTTACGAAATCTGTTGC
TGTTGAACCCTCTCCTGCAACTCTGCGATGCATTCAAGACAAATATCTTCAAAAACAGCATTTACAGG
TTTTTAAGATTGCACTTCCTGAATTTTGCGATGCACCTGACCAGGAAAGTGTTGAAAAAGCAGGCCAA
GAGTTTGGTTATCCTTTTGTACTGAAAAGTAAAACATTGGCTTACGACGGTCGTGGAAATTACGTTGT
TCATCAACCATCTGAGATTCCTACTGCCATCAAAGCACTTGGTGATCGTCCGCTTTATGTTGAAAAGT
TCGTTCCTTTCTCCATGGAAATTGCAGTGATGGTAGTACGCAGTTTAGACGGAAAAGTTTATGCTTAT
CCTACAACTGAGACCATTCAAAAGGATAATGTTTGTCATTTAGTATATGCCCCTGCTCGTCTTCCCTT
CTCAATTCAACAGCGTGCTCAAACCCTTGCCATGGATGCGGTGCGCACTTTTGAAGGCGCTGGTATA
TATGGTGTAGAGATGTTTGTTTTGCAAGATGGTGAGACCATTTTACTCAACGAAATTGCTCCTCGGCC
TCACAATTCAGGTCACTACACCATTGAAGCTTGCCCAACTTCTCAGTTTGAAGCTCACTTACGGGCCA
TATGTGGTCTTCCTTTCAGCGAAATCAACACCCAACTCTCGACTTCCACAACTCATGCGTTGATGGTA
AATATTTTAGGTACTGATGATCCTGATTATGTTTCAAAGATCGCTAAACGTTCTCTGTCCATTCCCGGT
GCAACTTTGCATCTTTATGGTAAAGCTGAATCTAGAAAGGGTCGCAAGATGGGACACGTTACCATCA
TTTCTGATTCACCTCAAGAATGTGAACGTAGGTATCAGATGCTTCTTGACGTCAAAGATCCTGTCGAA
TCACCTGTTGTTGGTATTATCATGGGTTCGGATTCTGATTTAAGCAAGATGAAAGATGCTGCCGTCAT
TTTAGATGAATTCAAGGTGCCTTACGAACTTACTATTGTTTCAGCTCACCGCACACCAGATCGCATGG
TTACTTATGCTCGTACCGCAGCTTCAAGAGGGTTGCGTGTGATTATTGCTGGTGCTGGTGGTGCCGC
TCATTTGCCTGGTATGGTTGCTGCAATGACACCTCTTCCAGTAATCGGCGTTCCTGTAAAAGGAAGC
ACTCCACTGTGTGCCGAACTAGCAATTGTGAGCGGATAACAATTACTAGCAATTGTGAGCGGATAAC
AATTACTAGCAATTGTGAGCGGATAACAATTACTAGTTCCTTCACTATTTTAACATTTTCAACCCTCCC
TTTTGGCTGCTATGGAGAGCTTTATGGACAATATGAAAGAGATTGTTTTAGAAAAGGCTGATAAATTA
GAAAAAGTTGGTTGGAAAAATTATTCTGCATAG

***ade6-3099* (R1)**

ATGAGCGAAAAACAGGTTGTAGGGATCCTTGGAGGTGGTCAATTGGGCCGAATGATGGTAGAGGCA
GCCCATCGCTTAAACATCAAATGCATCATCTTGGATGCAGCAAATTCTCCTGCCAAACAAATTGATGG
AGGACGTGAGCACATTGATGCATCATTTACTGACCCCGATGCAATTGTTGAACTGTCTAAGAAGTGC
ACGTTATTAACAACTGAAATTGAGCATATTAACACTGATGCCTTGGCAGCCGTTACGAAATCTGTTGC
TGTTGAACCCTCTCCTGCAACTCTGCGATGCATTCAAGACAAATATCTTCAAAAACAGCATTTACAGG
TTTTTAAGATTGCACTTCCTGAATTTTGCGATGCACCTGACCAGGAAAGTGTTGAAAAAGCAGGCCAA
GAGTTTGGTTATCCTTTTGTACTGAAAAGTAAAACATTGGCTTACGACGGTCGTGGAAATTACGTTGT
TCATCAACCATCTGAGATTCCTACTGCCATCAAAGCACTTGGTGATCGTCCGCTTTATGTTGAAAAGT
TCGTTCCTTTCTCCATGGAAATTGCAGTGATGGTAGTACGCAGTTTAGACGGAAAAGTTTATGCTTAT
CCTACAACTGAGACCATTCAAAAGGATAATGTTTGTCATTTAGTATATGCCCCTGCTCGTCTTCCCTT
CTCAATTCAACAGCGTGCTCAAACCCTTGCCATGGATGCGGTGCGCACTTTTGAAGGCGCTGGTATA
TATGGTGTAGAGATGTTTGTTTTGCAAGATGGTGAGACCATTTTACTCAACGAAATTGCTCCTCGGCC
TCACAATTCAGGTCACTACACCATTGAAGCTTGCCCAACTTCTCAGTTTGAAGCTCACTTACGGGCCA
TATGTGGTCTTCCTTTCAGCGAAATCAACACCCAACTCTCGACTTCCACAACTCATGCGTTGATGGTA

AATATTTTAGGTACTGATGATCCTGATTATGTTTCAAAGATCGCTAAACGTTCTCTGTCCATTCCCGGT
GCAACTTTGCATCTTTATGGTAAAGCTGAATCTAGAAAGGGTCGCAAGATGGGACACGTTACCATCA
TTTCTGATTCACCTCAAGAATGTGAACGTAGGTATCAGATGCTTCTTGACGTCAAAGATCCTGTCGAA
TCACCTGTTGTTGGTATTATCATGGGTTCGGATTCTGATTTAAGCAAGATGAAAGATGCTGCCGTCAT
TTTAGATGAATTCAAGGTGCCTTACGAACTTACTATTGTTTCAGCTCACCGCACACCAGATCGCATGG
TTACTTATGCTCGTACCGCAGCTTCAAGAGGGTTGCGTGTGATTATTGCTGGTGACTAGCAATTGTG
AGCGGATAACAATTTGGTTGCTGCAATGACACCTCTTCCAGTAATCGGCGTTCCTGTAAAAGGAAGC
ACTCTTGACGGAGTTGACTCTCTTCACTCTATTGTTCAGATGCCTCGAGGTGTCCCTGTCGCCACTG
TTGCTATCAATAATAGCCAAAATGCCGGTATTTTAGCCTGTCGTATACTTGCTACATTTCAACCCTCCC
TTTTGGCTGCTATGGAGAGCTTTATGGACAATATGAAAGAGATTGTTTTAGAAAAGGCTGATAAATTA
GAAAAAGTTGGTTGGAAAAATTATTCTGCATAG

Table S5. **Recombination with each LinE-LacI fusion and various *lacO* arrays**

ade6 allele [*lacO* position(s)]	*ade6-52*, LinE-LacI-NLS-3FLAG (+/+)[a]				
	No Fusion (-/-)	*mug20-231*	*rec10-233*	*rec25-230*	*rec27-232*
3098 (L1)	185 ± 35 (5)	369 ± 8 (3)	200 (1)	50 ± 8 (3)	206 ± 18 (4)
3102 (L3)	121 ± 18 (5)	502 ± 82 (5)		113 (1)	267 ± 37 (5)
3101 (L8)	89 ± 13 (8)	529 ± 27 (8)	200 (1)	60 (1)	158 ± 27 (5)
3099 (R1)	87 ± 11 (5)	220 ± 4 (2)	94 (1)	42 ± 3 (3)	138 ± 45 (3)
3111 (R8)	52 (1)	257 (1)			
M375	205 ± 9 (4)	208 ± 7 (4)	281 ± 25 (4)	109 ± 12 (4)	231 ± 18 (4)

[a] Recombinant frequency of *ade6::lacO* x *ade6-52* in crosses homozygous for the indicated LacI fusion. Data are Ade^+ per 10^6 viable spores [mean ± SEM in (n) crosses]. See Tables 1 – 5 and S6 for additional data.

Table S6. Recombination with Mug20-LacI fusions and various *lacO* arrays

ade6 allele [*lacO* position(s)]	*ade6-52, mug20* allele (+/+)[a]			
	No Fusion (-/-)	*mug20-231* (Mug20-LacI-NLS-3FLAG)	*mug20-252* (LacI-Mug20)	*mug20-254* (Mug20-LacI')
3098 (L1)	185 ± 35 (5)	369 ± 8 (3)		
3102 (L3)	121 ± 18 (5)	502 ± 82 (5)		
3101 (L8)	89 ± 13 (8)	529 ± 27 (8)	713 ± 107 (5)	576 ± 106 (4)
3101 (L8) +3 mM IPTG	119 ± 18 (4)	732 ± 127 (4)	142 ± 18 (4)	798 ± 121 (4)
3099 (R1)	87 ± 11 (5)	220 ± 4 (2)		
3111 (R8)	52 (1)	257 (1)		
3106 (L3, R3)	11 ± 1 (5)	97 ± 4 (5)	74 (1)	
3106 (L3, R3) +3 mM IPTG	7 (1)		11 (1)	
M375	205 ± 9 (4)	208 ± 7 (4)	72 ± 4 (4)	

[a] Recombinant frequency of *ade6::lacO* x *ade6-52* in crosses homozygous for the indicated LacI fusion. Data are Ade^+ per 10^6 viable spores [mean ± SEM in (n) crosses]. See Tables 1 – 5 and S5 for additional data.

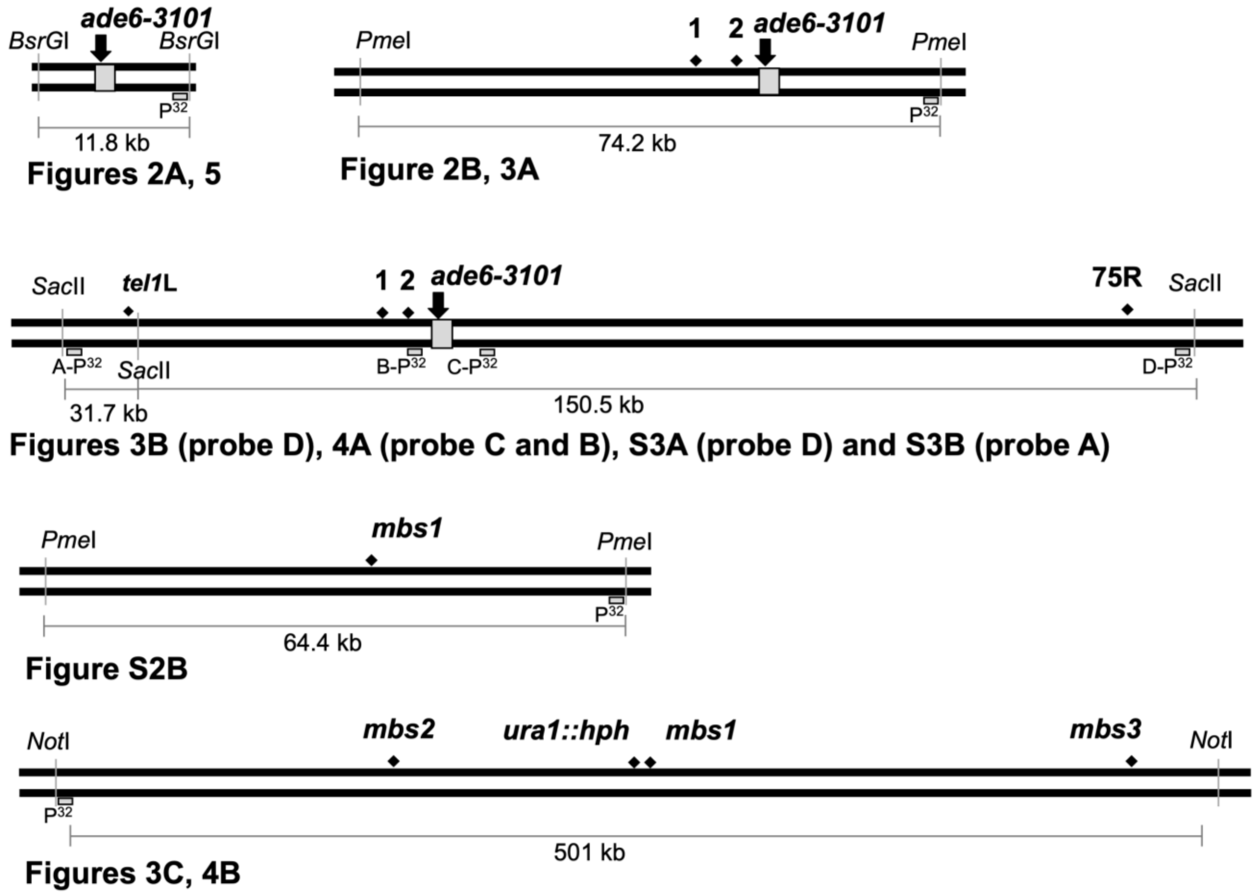

Figure S1. Probes and restriction enzymes used in Southern blot analysis. Meiotic DNA was digested with *BsrG*I (Figure 2A), *Pme*I (Figures 2B, 3A, and S2B), *Sac*II (Figures 3B, S3A and B), or *Not*I (Figures 3C and 4B) and probed with [P^{32}] DNA at the positions indicated. Note that probes are at the ends of the restriction fragments, except when double-cut DNA fragments were analyzed (Figure 4A and B); in those cases, internal DNA probes between the DSB hotspots of interest were used (probes B and C) to the left and right of *ade6*. The DNA is drawn to scale; the positions of DSB hotspots are indicated by black diamonds, except for *ade6-3101* (thick black arrow).

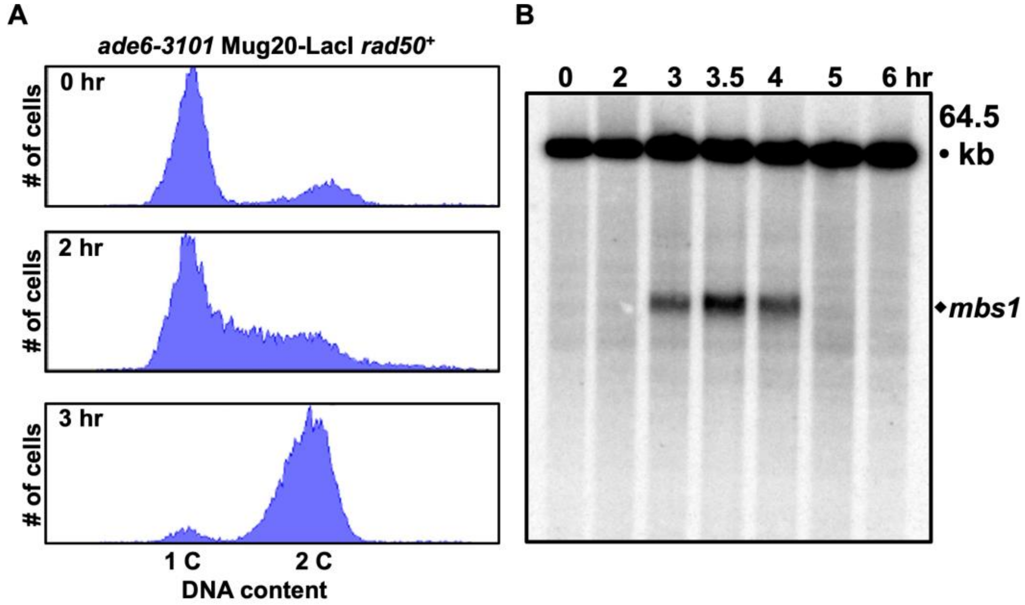

Figure S2. DSBs arise at the LinE-LacI-*lacO* hotspot before meiotic DNA replication. (**A**) Flow cytometry of strain GP9673 (*ade6-3101 mug20-231*) (10,000 cells at each time point) shows that replication started at about 2 hr, when DSBs at *ade6-3101* were first detected (Figure 2B). (**B**) DSBs at the endogenous hotpot *mbs1,* on a different chromosome, are not detected before replication. DNA was digested with *Pme*I and analyzed with a probe at the right end of the 64.4 kb fragment with *mbs1.*

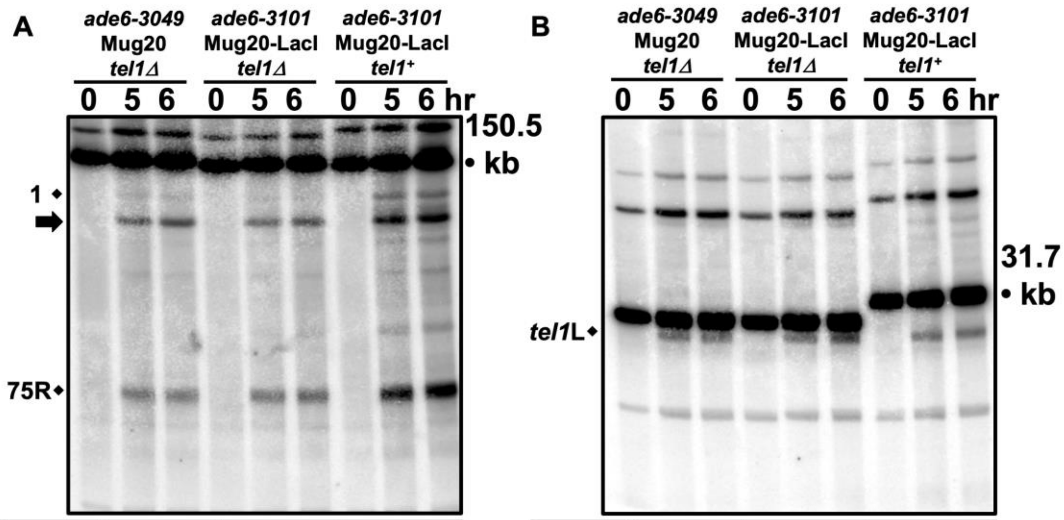

ade6	3.04 +/- 0.28%	3.56 +/- 0.13%	5.42 +/- 0.18%
75R	2.21 +/- 0.21%	3.75 +/- 0.13%	7.85 +/- 0.41%

*tel1*L	3.44 +/- 0.27%	3.73 +/- 0.11%	3.80 +/- 0.52%

Figure S3. Single-cut DSBs used to calculate the CoC of DSB hotspot pairs in Figure 4A. (**A**) As in Figure 3B, DNA was digested with *Sac*II and analyzed with a probe at the right end of the 150.5 kb fragment with *ade6*. Large arrow indicates prominent DSBs at *ade6-3101,* and small diamonds indicate lesser DSBs at hotspots 1 (15 kb to the left of *ade6*) and 75R (75 kb to the right of *ade6*). The product of the frequencies of each DSB was used to calculate the expected frequency of double-cut DSBs and the CoCs in Figure 4A. Quantification (mean ± SEM) is based on four blots from two independent inductions. (**B**) DNA was digested with *Sac*II; the probe was at the left end of the 31.7 kb fragment with *tel1* and the *tel1L* hotspot 2 kb to its left. In the *tel1Δ* strains, this DSB hotspot is caused by *natMX6*, substituting *tel1*. Quantification was as in **A**.

References

Boddy, M.N., Gaillard, P.-H.L., McDonald, W.H., Shanahan, P., Yates, J.R., and Russell, P. (2001). Mus81-Eme1 are essential components of a Holliday junction resolvase. Cell *107*, 537-548.

Cromie, G.A., Hyppa, R.W., Taylor, A.F., Zakharyevich, K., Hunter, N., and Smith, G.R. (2006). Single Holliday junctions are intermediates of meiotic recombination. Cell *127*, 1167-1178.

Davis, L., and Smith, G.R. (2003). Non-random homolog segregation at meiosis I in *Schizosaccharomyces pombe* mutants lacking recombination. Genetics *163*, 857-874.

Ellermeier, C., and Smith, G.R. (2005). Cohesins are required for meiotic DNA breakage and recombination in *Schizosaccharomyces pombe*. Proc. Natl. Acad. Sci. USA *102*, 10952-10957.

Farah, J.A., Cromie, G., Davis, L., Steiner, W.W., and Smith, G.R. (2005). Activation of an alternative, Rec12 (Spo11)-independent pathway of fission yeast meiotic recombination in the absence of a DNA flap endonuclease. Genetics *171*, 1499-1511.

Farah, J.A., Hartsuiker, E., Mizuno, K.-I., Ohta, K., and Smith, G.R. (2002). A 160-bp palindrome is a Rad50•Rad32-dependent mitotic recombination hotspot in *Schizosacchromyces. pombe*. Genetics *161*, 461-468.

Fowler, K.R., Hyppa, R.W., Cromie, G.A., and Smith, G.R. (2018). Physical basis for long-distance communication along meiotic chromosomes. Proc Natl Acad Sci U S A *115*, E9333-E9342.

Guerra-Moreno, A., Alves-Rodrigues, I., Hidalgo, E., and Ayte, J. (2012). Chemical genetic induction of meiosis in Schizosaccharomyces pombe. Cell Cycle *11*, 1621-1625.

Hyppa, R.W., and Smith, G.R. (2010). Crossover invariance determined by partner choice for meiotic DNA break repair. Cell *142*, 243-255.

Iino, Y., and Yamamoto, M. (1985). Mutants of *Schizosaccharomyces pombe* which sporulate in the haploid state. Molecular and General Genetics *198*, 416-421.

Kitajima, T.S., Yokobayashi, S., Yamamoto, M., and Watanabe, Y. (2003). Distinct cohesin complexes organize meiotic chromosome domains. Science *300*, 1152-1155.

Lin, Y., and Smith, G.R. (1995). Molecular cloning of the meiosis-induced *rec10* gene of *Schizosaccharomyces pombe*. Current Genetics *27*, 440-446.

Ma, L., Fowler, K.R., Martin-Castellanos, C., and Smith, G.R. (2017). Functional organization of protein determinants of meiotic DNA break hotspots. Sci Rep *7*, 1393.

Martin-Castellanos, C., Blanco, M., Rozalen, A.E., Perez-Hidalgo, L., Garcia, A.I., Conde, F., Mata, J., Ellermeier, C., Davis, L., San-Segundo, P.*, et al.* (2005). A large-scale screen in *S. pombe* identifies seven novel genes required for critical meiotic events. Current Biology *22*, 2056-2062.

Smith, G.R. (2009). Genetic analysis of meiotic recombination in *Schizosaccharomyces pombe*. In Meiosis, S. Keeney, ed. (Totowa, NJ: Humana Press), pp. 65-76.

Unnikrishnan, A., Akiyoshi, B., Biggins, S., and Tsukiyama, T. (2012). An efficient purification system for native minichromosome from Saccharomyces cerevisiae. Methods Mol Biol *833*, 115-123.
